# Locus-specific paramutation in *Zea mays* is maintained by a PICKLE-like chromodomain helicase DNA-binding 3 protein controlling development and male gametophyte function

**DOI:** 10.1371/journal.pgen.1009243

**Published:** 2020-12-15

**Authors:** Natalie C. Deans, Brian J. Giacopelli, Jay B. Hollick

**Affiliations:** 1 Department of Molecular Genetics, The Ohio State University, Columbus, Ohio, United States of America; 2 Centers for RNA Biology and Applied Plant Sciences, The Ohio State University, Columbus, Ohio, United States of America; University of Minnesota, UNITED STATES

## Abstract

Paramutations represent directed and meiotically-heritable changes in gene regulation leading to apparent violations of Mendelian inheritance. Although the mechanism and evolutionary importance of paramutation behaviors remain largely unknown, genetic screens in maize (*Zea mays*) identify five components affecting 24 nucleotide RNA biogenesis as required to maintain repression of a paramutant *purple plant1* (*pl1*) allele. Currently, the RNA polymerase IV largest subunit represents the only component also specifying proper development. Here we identify a chromodomain helicase DNA-binding 3 (CHD3) protein orthologous to Arabidopsis (*Arabidopsis thaliana*) PICKLE as another component maintaining both *pl1* paramutation and normal somatic development but without affecting overall small RNA biogenesis. In addition, genetic tests show this protein contributes to proper male gametophyte function. The similar mutant phenotypes documented in Arabidopsis and maize implicate some evolutionarily-conserved gene regulation while developmental defects associated with the two paramutation mutants are largely distinct. Our results show that a CHD3 protein responsible for normal plant ontogeny and sperm transmission also helps maintain meiotically-heritable epigenetic regulatory variation for specific alleles. This finding implicates an intersection of RNA polymerase IV function and nucleosome positioning in the paramutation process.

## Author summary

Genes are switched “on” and “off” during normal development by regulating DNA accessibility within the chromosomes. How certain gene variants permanently maintain “off” states from one generation to the next remains unclear, but studies in multiple eukaryotes implicate roles for specific types of small RNAs, some of which define cytosine methylation patterns. In corn, these RNAs come from at least two RNA polymerase II-derived complexes sharing a common catalytic subunit (RPD1). Although RPD1 both controls the normal developmental switching of many genes and permanently maintains some of these “off” states across generations, how RPD1 function defines heritable DNA accessibility is unknown. We discovered that a protein (CHD3a) belonging to a group known to alter nucleosome positioning is also required to help maintain a heritable “off” state for one particular corn gene variant controlling both plant and flower color. We also found CHD3a necessary for normal plant development and sperm transmission consistent with the idea that proper nucleosome positioning defines evolutionarily-important gene expression patterns. Because both CHD3a and RPD1 maintain the heritable “off” state of a specific gene variant, their functions appear to be mechanistically linked.

## Introduction

Organismal development requires alleles to undergo controlled transitions between silent and expressed states coordinated by transcription factors, non-coding RNAs, and chromatin regulators interacting with allele-specific regulatory sequences. In many eukaryotes, small non-coding RNAs (sRNAs) such as small interfering RNAs (siRNAs) (reviewed in [1]), microRNAs (miRNAs) (reviewed in [2]), and PIWI-interacting RNAs (piRNAs) (reviewed in [3])—in complex with argonaute (AGO) proteins—can modulate chromatin structure, RNA stability, or translation to achieve developmental transitions and/or defend against foreign nucleic acids and transposable element (TE) activities (reviewed in [1]). sRNAs are typically processed from DNA-dependent RNA polymerase (RNAP) II transcripts, but in multicellular plants, additional RNAP II-related complexes [4,5] having specialized functions in transcriptional regulation [6–9] generate the majority of siRNA precursors.

RNAP II-related RNAPs IV and V collaborate to maintain repressive chromatin states through the action of RNAP IV-derived siRNAs that primarily target TEs and other repetitive sequences for *de novo* cytosine methylation and subsequent histone modifications via a process termed RNA-directed DNA Methylation (RdDM) [10]. Although Arabidopsis (*Arabidopsis thaliana*) plants lacking RNAP IV and/or V develop normally [11], misexpression of specific alleles in the absence of the maize (*Zea mays*) RNAP IV largest subunit (RPD1) leads to abnormal development [7,8,12–14]. Thus, in maize and likely other grasses, RNAP IV has been co-opted to define some developmental expression patterns. Unlike the eudicots typified by Arabidopsis, grasses have diversified subtypes for RNAP IV, V, and outside of maize, RNAP VI [15] defined by alternative second largest catalytic subunits [5]. The evolutionary importance of such diversity and regulatory novelty remains completely unknown.

Maize RNAP IV subtypes also establish and/or maintain meiotically heritable expression patterns of certain alleles [13,16–19] for which changes in gene regulation occur in response to *trans*-homolog interactions by a process known as paramutation [20–23]. Such alleles are well described at *booster1* (*b1*) [24–27] and *red1* (*r1*) [20,28,29]—loci encoding basic helix-loop-helix (bHLH) proteins—and at both the *pericarp color1* (*p1*) [30–32] and *purple plant1* (*pl1*) [33] loci encoding R2R3 Myb-type proteins. Expression patterns of these alleles can be directly visualized by pigmentation [34] because the encoded proteins are transcriptional activators of genes encoding flavonoid biosynthetic enzymes. Similar to developmental phase changes [35], certain alleles of these loci can switch from transcriptionally active to repressed states [36–38].

Characteristic of paramutation, the repressed state of a given allele appears dominant to an active one, and typically only repressed states are sexually transmitted from such heterozygotes [22,23]. Hence, active states heritably change in response to being heterozygous with a homologous allele in a repressed state. Mutation screens identify loci that function as *mediators of paramutation* (*mop*) of the *B1-Intense* (*B1-I*) allele [18,39] and factors *required to maintain repression* (*rmr*) of the *Pl1-Rhoades* allele [40,41]. All five known MOP and RMR proteins are either RNAP IV subunits [13,17–19] or accessory proteins [41–43] required for 24 nucleotide (24nt) RNA biogenesis [6,13,17,18,43–45].

The involvement of small RNA biogenesis components in facilitating and/or maintaining paramutations implicates a model in which regulatory landscapes are transferred between homologous alleles with differing epigenetic states via 24nt RNAs shared in *trans* [22,23,46]. Curiously, no potential components of an RdDM-type pathway downstream of 24nt RNAs have yet been identified in the *mop* and *rmr* screens. Two RNAP IV catalytic subunits encoded by *rmr6* / *mop3* / *rpd1* [13,19] and *rmr7* / *mop2* / *rpd2a* [17,18] are orthologs of Arabidopsis NUCLEAR RNA POLYMERASE D1 (NRPD1) and NRPD2, respectively. Additionally, *mop1* encodes a likely RNA-dependent RNA polymerase (RDR) orthologous to Arabidopsis RDR2 [42], and *rmr1* encodes an SNF2-type ATP-dependent helicase similar to Arabidopsis CLASSY 3 and 4 [41]. The novel RMR2 protein is also required for full 24nt RNA biogenesis [43] but functions of any Arabidopsis orthologs remain uncharacterized. Outside of stochastic defects reported for some *mop1* mutants [39], only loss of RPD1 persistently impacts plant development [12,13], indicating that RNAP IV has a role in developmental gene control independent of 24nt RNAs and any RdDM-type mechanism.

Paramutation-like behaviors in several non-plant species (reviewed in [23,47]) involve diverse sRNA-dependent mechanisms. In maize, a model that 24nt RNAs facilitate paramutations does not account for the observations that RMR1, RMR2, and RPD2a are not required to establish paramutations at *Pl1-Rhoades* [17,41,43]. These data indicate that although 24nt RNAs are implicated in maintaining *Pl1-Rhoades* repression, they may not be paramutation instigators, and the role of RNAP IV in facilitating paramutations may be independent of 24nt RNA biogenesis [6,8].

Here we describe a novel *rmr* locus (*rmr12*) where loss of function broadly affects plant development in ways mostly distinct from that of *rpd1* mutants. Mutations, genetic mapping, and sequence information show *rmr12* corresponds to a gene encoding a chromodomain helicase DNA-binding 3 (CHD3) protein most orthologous to Arabidopsis PICKLE (PKL). Genetic experiments show this CHD3 protein operates both somatically and in male gametophytes to ensure proper development and gamete transmission respectively. Small RNA profiling shows that RMR12 is not a component of 24nt RNA biogenesis yet genetic tests show that it specifically maintains *Pl1-Rhoades* repression and contributes to fidelity of the heritable feature(s) underlying its paramutagenic properties. Hence a likely nucleosome remodeler is responsible for specifying both mitotically- and meiotically-heritable epigenetic information.

## Results

### Mutations define the *rmr12* locus

**Fig 1:**
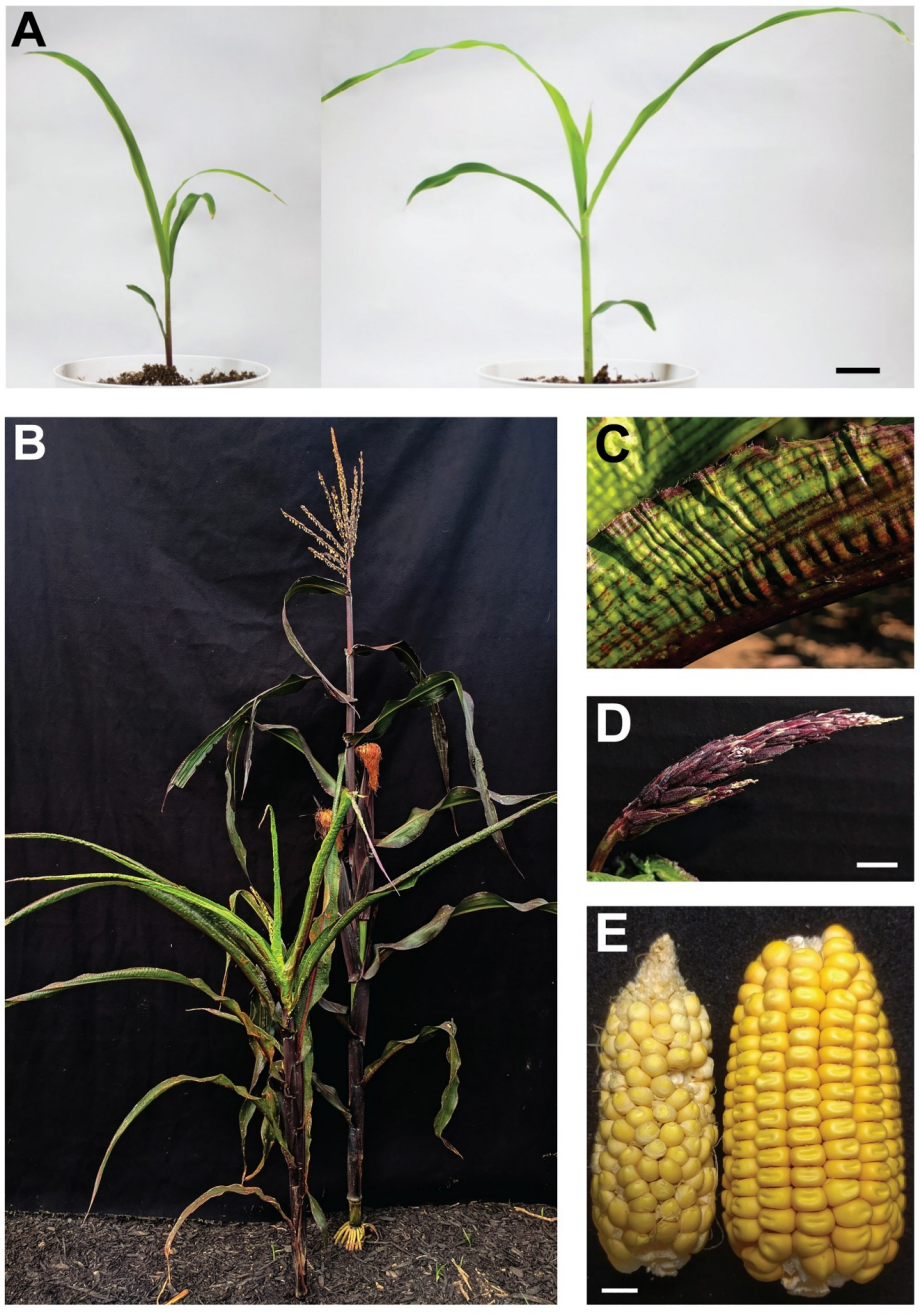
Mutant phenotypes. **(A)** Comparison of ems063095 mutant (left) and non-mutant sibling (right) seedlings. Bar = 2cm. **(B)** Comparison of adult ems063095 mutant (left) and flowering non-mutant sibling (right). **(C)** Abaxial surface of adult ems063095 mutant leaf blade. **(D)** ems063095 mutant tassel at flowering. Bar = 1cm. **(E)** Comparison of grain set on ems98738 mutant (left) and A619 (right) generated from reciprocal crosses. Bar = 1cm.

Because repressed *Pl1-Rhoades* states invariably condition weak pigmentation, mutations disrupting functions required to maintain this repression are easily identified by increased anthocyanin production [40]. Two distinct *rmr* screens using ethyl methanesulfonate (ems)-treated pollen [40,41] identified four mutations that also conferred similar developmental defects. Mutations ems98738 and ems98924 conditioned dark seedling pigmentation [40] while ems063095 and ems143190 were found with strongly pigmented anthers [41]. In all M_2_ and F_2_ progenies, dark seedling pigmentation exclusively cosegregated with narrow leaves and acute leaf angles (Fig 1). At maturity, all mutants had a dwarf stature, delayed flowering, and upright, narrow, adaxially-curled leaves (Fig 1B) having a wrinkled epidermis (Fig 1C, see S1 Fig). Inflorescences were either absent, barren, or small, with tassels having fewer secondary spikes and florets that rarely extruded anthers (Fig 1D). Grain set was rare with cobs carrying few and heterogeneous sized kernels set in disorganized rows (Fig 1E). In two F_2_ progenies segregating ems063095, all plants having fully-pigmented anthers diagnostic of the phenotype conferred by *Pl1-Rhoades* in a derepressed state (Pl-Rh) had the same developmental defects including later flowering and reduced height compared to their normal siblings displaying anther color phenotypes conditioned by a repressed *Pl1-Rhoades* state (Pl´) (Table 1). In these and all other examples, the function identified by these four mutations specified both proper plant development and apparent *Pl1-Rhoades* repression.

**Table 1:** Characters of ems063095 F_2_ individuals having Pl´ and Pl-Rh anther types.

	Pl´			Pl-Rh		
Progeny ID	DTF	Plant height	n	DTF	Plant height	n
091133	67 ±0.6	184.0 ±1.7	92	90 ±1.5	102.5 ±2.7	13
091061	65 ±0.5	212.5 ±1.7	72	92 ±2.6	127.8 ±6.2	6
Total	66 ±0.4	196.5 ±1.6	164	90 ±1.3	110.5 ±3.8	19

Mean ±s.e.m.; DTF: Days to flowering

Genetic complementation test results showed all four mutations define a novel *rmr* locus (see S1 and S2 Tables) hereafter designated *rmr12* with ems98738, ems98924, ems063095 and ems143190 mutations respectively renamed *rmr12-1*, *-2*, *-3*, and *-4*. For all four alleles, the mean frequency of mutant types was less than expected from single-locus recessive mutations (Tables 2 and 3, see S1 Methods, S3 Table). For instance, the frequency of *rmr12-1* and *rmr12-2* darkly-pigmented M_2_ seedlings was 0.10 (*χ*^2^ = 9.0, *p* = 0.0027) and 0.13 (*χ*^2^ = 8.6, *p* = 0.0033) respectively (Table 2), and the combined mutant frequency in 36 F_2_ progenies segregating *rmr12-1*, *rmr12-2*, or *rmr12-3* was 0.18 (Table 3, see S3 Table). These observations indicate the mutations are fully recessive. Comparing the observed and expected mutant frequencies defined by a single-locus (0.25) versus a two-locus model (0.0625) generated highly significant *χ*^2^ values of 61.02 and 493.08, respectively (Table 3). Although these results support neither model, the most parsimonious interpretations are that either transmission of single-locus recessive mutant alleles is impaired or that the mutant phenotype is incompletely penetrant.

**Table 2:** M_2_ mutant seedling segregation.

Allele	Progeny ID	No. dark seedlings	Total seedlings	Frequency	*p* value (χ^2^)
*rmr12-1*	98738	10	100	0.10	0.0027
*rmr12-2*	98924	18	142	0.13	0.0033

**Table 3:** F_2_ *rmr12* mutant frequencies.

Progeny		No. individuals			Statistics for single locus model (0.25)		Statistics for two locus model (0.0625)	
No.	Allele	Mutant	Non-mutant	Freq.	Mutant χ^2^	*p* value	Mutant χ^2^	*p* value
18	*rmr12-1*	111	642	0.15	31.70	9.3e-9	86.86	5.9e-21
2	*rmr12-2*	25	171	0.13	11.76	3.3e-4	13.27	1.4e-4
15	*rmr12-3*	303	1292	0.19	22.99	8.5e7	414.66	1.8e-92
	Total	439	2105	0.18	61.02	2.9e-15	493.08	1.5e-109

### Biased allele transmission is due to male gametophyte dysfunction

Because heterozygotes for *rmr12* mutant alleles bear cobs with near full grain set (see S2 Fig) we inferred that mutant female gametophytes were fully functional, and therefore surmised that mutant male gametophytes were compromised. To address this idea, we reciprocally crossed *rmr12-1 / rmr12-1* mutants to or by *rmr12-3 / Rmr12* heterozygotes looking for paternal transmission bias. Half the offspring had developmental defects (Fig 1) when *rmr12-3 / Rmr12* heterozygotes were used as females (0.49, *χ*^2^ = 0.10, *p* = 0.747), indicating that mutant sporophyte germination and survival are not impaired and that the mutant phenotype is fully penetrant (Table 4). In contrast, fewer mutants were observed when *rmr12-3 / Rmr12* heterozygotes were used as males (0.39, *χ*^2^ = 5.28, *p* = 0.021) (Table 4). Because all four independent *rmr12* mutant alleles show similar transmission ratio distortions (Tables 2 and 3, see S2 and S3 Tables), we hypothesized that *Rmr12* is important for normal male gametophyte function.

**Table 4:** Mutant frequencies in progeny from reciprocal crosses.

Parents		Progeny				
Female	Male	ID	Mutants	Non-mutants	Freq.	*p* value (χ^2^)
*rmr12-3* / *Rmr12*	*rmr12-1* / *rmr12-1*	142783	84	90	0.483	0.75
		142785	66	68	0.493	0.90
*rmr12-1* / *rmr12-1*	*rmr12-3* / *Rmr12*	142763	60	86	0.410	0.13
		142850	21	42	0.333	0.06
*rmr12-3* / *Rmr12*	*rmr12-1* / *rmr12-1*	Totals	150	158	0.487	0.75
*rmr12-1* / *rmr12-1*	*rmr12-3* / *Rmr12*		81	128	0.388	0.02

Because we observed genetic linkage of *rmr12* to a mutant *waxy1* (*wx1*) allele (see S1 Methods and S4 Table), we could test this hypothesis using *9S* cell autonomous markers to monitor *rmr12* allele transmissions. Because *wx1* mutant pollen accumulate amylopectins that stain red with I_2_-KI [48] rather than amylose which stains blue, we could approximate the frequency of *rmr12* alleles segregated from heterozygous plants. Fresh pollen collected from two *Rmr12 wx1 / rmr12-3 Wx1* individuals were of two types (1392 blue and 1350 red) whose frequencies did not deviate from the expected 0.50 (*χ*^*2*^ = 0.37, *p* = 0.57) (Table 5). The frequency of viable pollen as assessed with fluorescein diacetate [49] was also similar between *Rmr12* / *rmr12-4* and *Rmr12* / *Rmr12* individuals (0.97 in both, *p* = 0.94, two-sample *t*-test, see S3A Fig). We then compared *in vitro* pollen germination frequencies from eight *Rmr12 wx1 / rmr12-3 Wx1* florets. While frequencies varied from 0.3 to 0.6, the ratio of *Wx1* to *wx1* germinated pollen from each floret did not significantly differ from 1 (*p* = 0.64, one-sample *t*-test; see S3B Fig). These data indicate that the *rmr12* mutations transmission biases are not due to meiotic errors, grain filling defects or failed pollen germination.

**Table 5:** Frequency of I_2_-KI stained pollen types from *Rmr12 wx1 / rmr12-3 Wx1* individuals.

Progeny ID	Individual	Blue pollen	Red pollen	Red frequency	*p* value (χ^*2*^)
160372	17-62-5	945	873	0.48	0.23
160372	17-62-8	477	447	0.52	0.49
Total		1392	1350	0.51	0.57

We next evaluated paternal *rmr12* allele transmissions via their linkage to *colored aleurone1* (*c1*), a locus required for kernel pigmentation [50,51] located approximately 30 cM from *wx1* [52,53]. We crossed *Rmr12 c1 / rmr12-4 C1* males to recessive *c1* testers and recorded both the frequency and distribution of colored kernels on each testcross cob. Because the mean frequency of colored kernels from all cobs (0.43) was significantly less than the expected 0.50 (*p* = 6.34e-07, one-sample *t*-test) (Table 6), we concluded that *C1* transmission reflects that of *rmr12-4*. Furthermore, because there was no indication of aborted ovules (see S2B Fig), the transmission bias appeared to occur prior to fertilization. To test whether the bias was possibly due to differential pollen tube growth, we compared *C1* transmission in the apical half of the cob to that in the basal half (Table 6) where pollen tubes would be longer. Colored kernel mean frequencies, 0.44 (apical) vs 0.42 (basal), did not differ (*p* = 0.22, two-sample *t*-test) indicating that the allele transmission bias is not due to obvious pollen tube growth competitions. Pollen tube lengths of *in vitro* germinated pollen from 8 *Rmr12 wx1* / *rmr12-4 Wx1* florets were also no different between the two stained types (*p* = 0.27, two-sample *t*-test; see S3C Fig). From these results, we conclude that *rmr12* mutant pollen grains are not compromised in viability, germination, or pollen tube growth but have an unknown and incompletely penetrant male gametophyte defect.

**Table 6:** Cob position of testcrosses kernel phenotypes representing transmission of *rmr12-4* linked *c1* alleles.

	Basal half			Apical half			Total			
Progeny ID	C1	c1	Freq.	C1	c1	Freq.	C1	c1	Freq.	*p* value (χ^2^)
170107	73	90	0.45	68	99	0.41	141	189	0.42	0.06
170118	67	73	0.48	66	84	0.44	133	157	0.46	0.32
170122	63	86	0.42	56	72	0.43	119	158	0.43	0.10
170124	57	86	0.40	42	44	0.49	99	130	0.43	0.15
170131	56	84	0.40	50	54	0.48	106	138	0.43	0.15
170134	91	100	0.48	63	74	0.46	154	174	0.47	0.43
170323	100	193	0.34	97	121	0.44	197	314	0.39	0.0002
170324	59	74	0.44	58	82	0.41	117	156	0.43	0.09
170782	29	51	0.36	45	64	0.44	74	115	0.39	0.03
Total	595	837	0.42	545	694	0.44	1140	1531	0.43	4.2e-8

### The *rmr12* locus encodes a CHD3 protein

To better understand the link between development, gametophyte function and *Pl1-Rhoades* repression, we identified the molecular nature of *rmr12* using positional information and sequence analysis. A BC_3_F_2_ mapping population was developed between the mutagenized A619 and recurrent A632 parental lines and, because *wx1* and *c1* linkages confirmed a *9S* position, *rmr12-3* mutants were genotyped with polymorphic *9S* molecular markers (Table 7). This analysis narrowed the lesion to a 4Mb interval having 109 gene models (see Methods and S5 Table), none of which encode obvious RdDM-related proteins. One model, however, encodes a chromatin-related protein, a putative member of the homeodomain-like transcription factor superfamily (Zm00001d045109, *chr113*) composed of a plant homeodomain zinc-finger (PHD), tandem chromodomains, bipartite SNF2-type helicase, and two conserved domains of unknown function (DUF 1086 and 1087) diagnostic of chromodomain helicase DNA-binding 3 (CHD3) proteins [54].

**Table 7:** Recombination-based mapping of *rmr12-3* from F_2_ mutants.

Molecular Marker			No. individuals with specified genotype			
ID	Type	Chr *9* location^a^	A619 / A619	Heterozygotes	A632 / A632	% A619
9_12.38	CAPS	12,377,322	66	2	0	99%
9_16.47	dCAPS	16,471,870	25	9	1	84%
umc1586	SSR	25,251,065	16	16	3	69%
umc2128	SSR	25,743,586	4	4	1	67%
9_50.02	CAPS	50,997,919	6	5	1	71%
9_61.09	dCAPS	70,090,283	15	13	3	69%
9_87.31	CAPS	89,637,894	7	5	3	63%
umc1267	SSR	105,956,029	0	0	20	0%

^**a**^B73 AGPv4 genome

**Fig 2:**
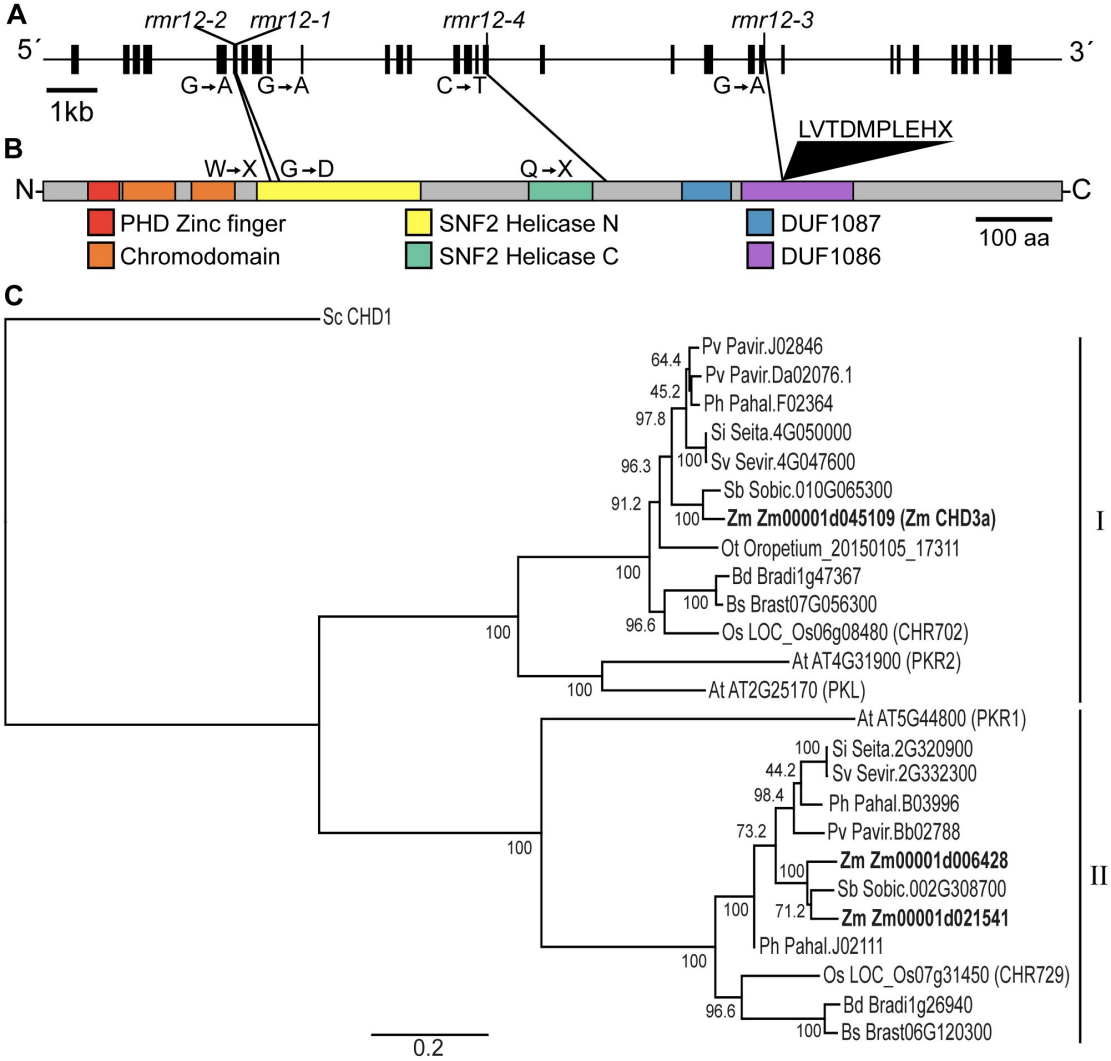
*rmr12* mutations disrupt a CHD3-encoding gene model. **(A)** Maize gene model Zm00001d045109 highlighting transition mutations found in cDNA sequences from *rmr12-1*, *rmr12-2*, *rmr12-3*, and *rmr12-4* mutants. **(B)** Predicted protein model highlighting domains diagnostic of CHD3 proteins and missense, nonsense (X), and insertion lesions corresponding to the respective transition mutations in **(A)**. **(C)** Maximum likelihood tree produced from alignment of full-length maize (Zm) CHD3 protein sequences with CHD3 proteins from Arabidopsis (At) and grasses including *Brachypodium distachyon* (Bd), *Brachypodium stacei* (Bs), *Oropetium thomaeum* (Ot), rice (Os), *Panicum hallii* (Ph), *Panicum virgatum* (Pv), *Setaria italica* (Si), *Setaria viridis* (Sv), and *Sorghum bicolor* (Sb) identifies two clades (I and II). The tree is anchored with *Saccharomyces cerevisiae* (Sc) CHD1. Branch lengths depict substitutions per site.

*rmr12-3* mutant cDNA sequence (see S4 and S5 Figs) revealed a transition-type mutation in Zm00001d045109 (Fig 2A) that eliminated a canonical intron splice acceptor site and identified 67nt of retained intron sequence unique from the 37 transcript isoforms predicted in the B73 AGPv4 transcriptome [55]. All tested *rmr12-3* mutants (*n* = 33) were homozygous for this mutation as identified with a dCAPS marker. This retained intron isoform encodes nine additional amino acids and a premature stop codon (Fig 2B; see S5 and S6 Figs). Sequences of tiled amplicons spanning the longest Zm00001d045109 transcript isoforms in all *rmr12-1*, *rmr12-2*, *rmr12-3*, and *rmr12-4* mutant and non-mutant cDNAs supported Zm00001d045109_T004 as a predominant transcript and identified additional unique transition mutations (Fig 2A; see S5 Fig). A G to A missense in *rmr12-1* changes glycine 308 to aspartic acid, and independent nonsense mutations were found in *rmr12-2* and *rmr12-4* (Fig 2B; see S5 and S6 Figs). Because of the *rmr12-4*-associated mutation, any translated protein would lack both DUF domains that in CHD3 proteins may bind DNA [56]. The G308D—occurring within an invariant GK(T/S) sequence of motif I where the adjacent lysine coordinates either a gamma or beta ATP phosphate in all SNF2-type ATPases [57]—would negatively affect ATP-binding, and any *rmr12-2*-encoded protein would lack all but the PHD and chromodomains (see S6 Fig). These coincident and disruptive lesions found in the defined *9S* interval of plants homozygous for each of the four *rmr12* mutant alleles strongly indicates that the *rmr12* locus consists of a gene (Zm00001d045109) encoding a CHD3 protein, a subgroup of CHD proteins with known roles in transcriptional regulation [58].

A tBLASTn analysis of the B73 AGPv4 genome provided no evidence of a Zm00001d045109 duplicate but did identify closely related gene models (Zm00001d006428 and Zm00001d021541) in syntenic *2L* and *7L* regions. RNA-seq reads from all three *chd3* genes are detected in each of 23 developmental and reproductive tissues including pollen [59] though Zm00001d045109 reads are most abundant (5–10 fold greater) in all datasets. A phylogenetic comparison of plant proteins having the DUF 1087 region, the most exclusive and conserved feature within the CHD3 clade, shows that, similar to eudicots and other grasses [60,61], maize has two distinct CHD3 subfamilies (Fig 2C). Each surveyed species has at least one subfamily I member homologous to the Zm00001d045109-encoded CHD3 that clades most closely with Arabidopsis PKL, whereas the other maize CHD3 proteins belong to the Arabidopsis PICKLE RELATED 1 (PKR1) clade II and appear to have arisen through a maize-specific whole genome duplication [62]. Zm00001d045109 is hereafter referred to as *chd3a*.

### *Rmr12* is required for normal development

**Fig 3:**
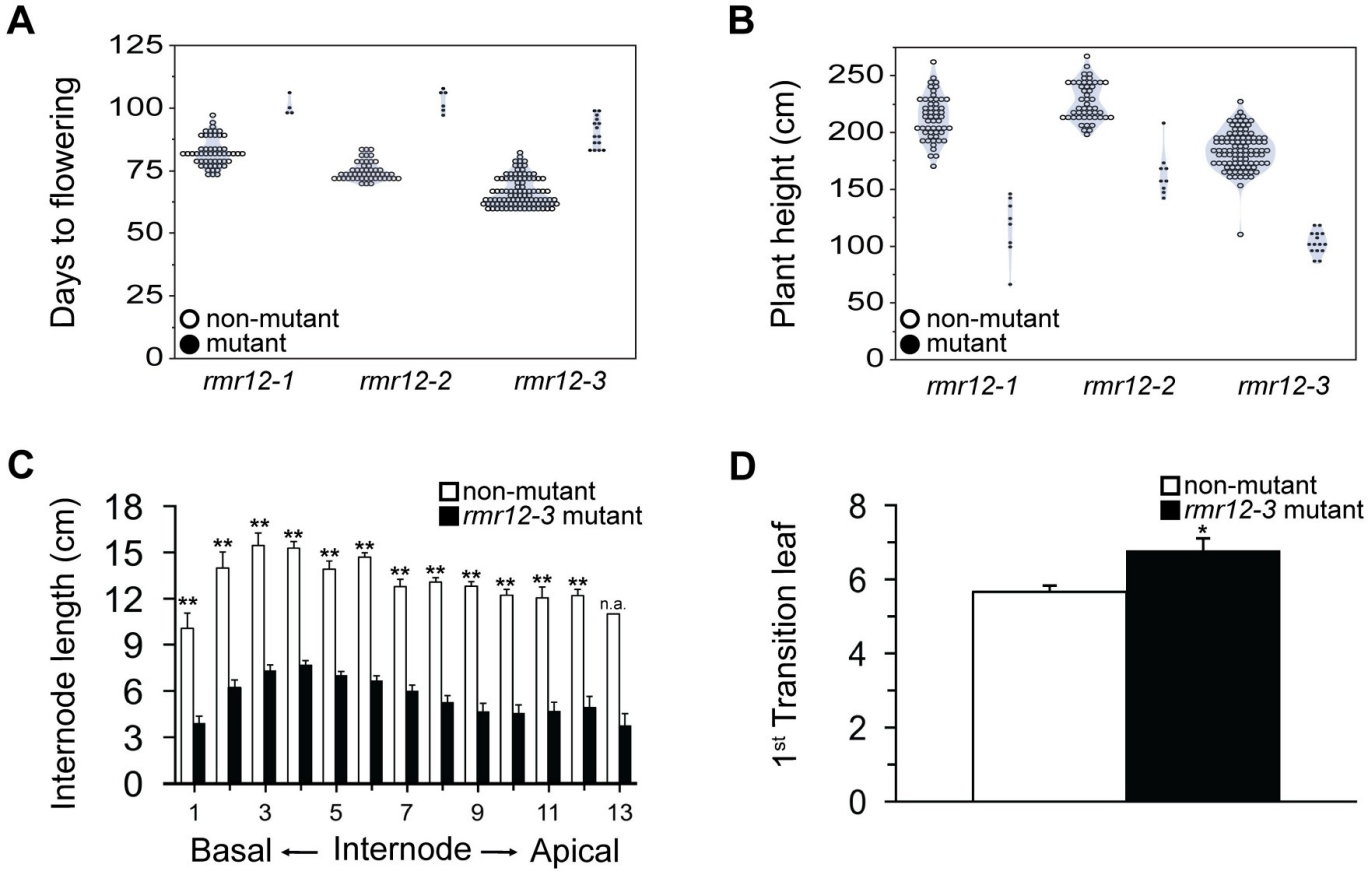
Developmental profiles of *rmr12* mutant and non-mutant F_2_ siblings. **(A)** Days to flowering for individual progenies segregating homozygotes for *rmr12-1* (mutant n = 4, non-mutant n = 53, *p* = 0.0009), *rmr12-2* (mutant n = 6, non-mutant n = 50, *p* = 5.04e-6), or *rmr12*-3 (mutant n = 15, non-mutant n = 98, *p* = 9.00e-12). **(B)** Plant heights at flowering for progenies described in **(A)**, *rmr12-1* (mutant n = 8, non-mutant n = 54, *p* = 9.86e-6), *rmr12-2* (mutant n = 9, non-mutant n = 50, *p* = 3.89e-6), or *rmr12-3* (mutant n = 15, non-mutant n = 98, *p* = 5.07e-22). **(C)** Mean internode lengths (±s.e.m.) for one progeny segregating *rmr12-3* homozygotes (mutant n = 12, non-mutant n = 12). ** *p*<0.001, n.a. = not available (single value). **(D)** Mean first leaf (±s.e.m.) with adult-type leaf waxes for 9 *rmr12-3* mutants and 9 non-mutants from a single progeny. * *p* = 0.01.

In addition to *pkl* mutants developing pickle-like root structures [63], they have reduced plant height [64], delayed vegetative phase change [65] and flowering [64], and reduced floral structures with aborted ovules [66]. These defects are similar to those displayed in *rmr12* mutants (Fig 1) consistent with *pkl* and *rmr12* providing orthologous functions. In contrast, many *rmr12* mutant developmental defects appear distinct from those of *rpd1* mutants [12]. These observations motivated a more detailed analysis to better define these similarities and differences. Quantifying days to flowering (DTF) and plant heights of independent F_2_ progenies segregating *rmr12-1*, *rmr12-2*, or *rmr12-3* homozygotes (Fig 3A and 3B), we found all mutants flowered significantly later with a mean increase of 23.4 days (+/- 2.8 s.e.m., *p* = 0.0009, 5.04e-6, and 9.00e-12, two-sample *t*-test) and were shorter, on average 0.39 times the non-mutant sibling heights (+/- 0.06 s.e.m., *p* = 9.86e-6, 3.89e-6, and 5.07e-22, two-sample *t*-test). We conclude, therefore, that *Rmr12* governs fundamental processes affecting plant growth.

Because the *rpd1* mutant dwarf stature is exclusively due to reduced adult-phase internode lengths [12], we compared F_2_ *rmr12-3* mutant and non-mutant sibling internode lengths at flowering. Although average mutant leaf number was greater than that of non-mutant siblings (12.7 versus 11.3 respectively; *p* = 0.003, two-sample *t*-test), all internode lengths, including those of the juvenile-phase, were significantly shorter (Fig 3C). Because the *rpd1* mutant juvenile to adult phase transition is also delayed [12], we compared the average first leaf displaying adult leaf waxes (see Methods) between F_2_ *rmr12-3* mutants and non-mutant siblings. Similar to *rpd1* mutants, the *rmr12-3* mutant transition was delayed by 1.1 leaves (leaf 5.7 vs leaf 6.8, *p* = 0.01, two-sample *t*-test) (Fig 3D).

**Fig 4:**
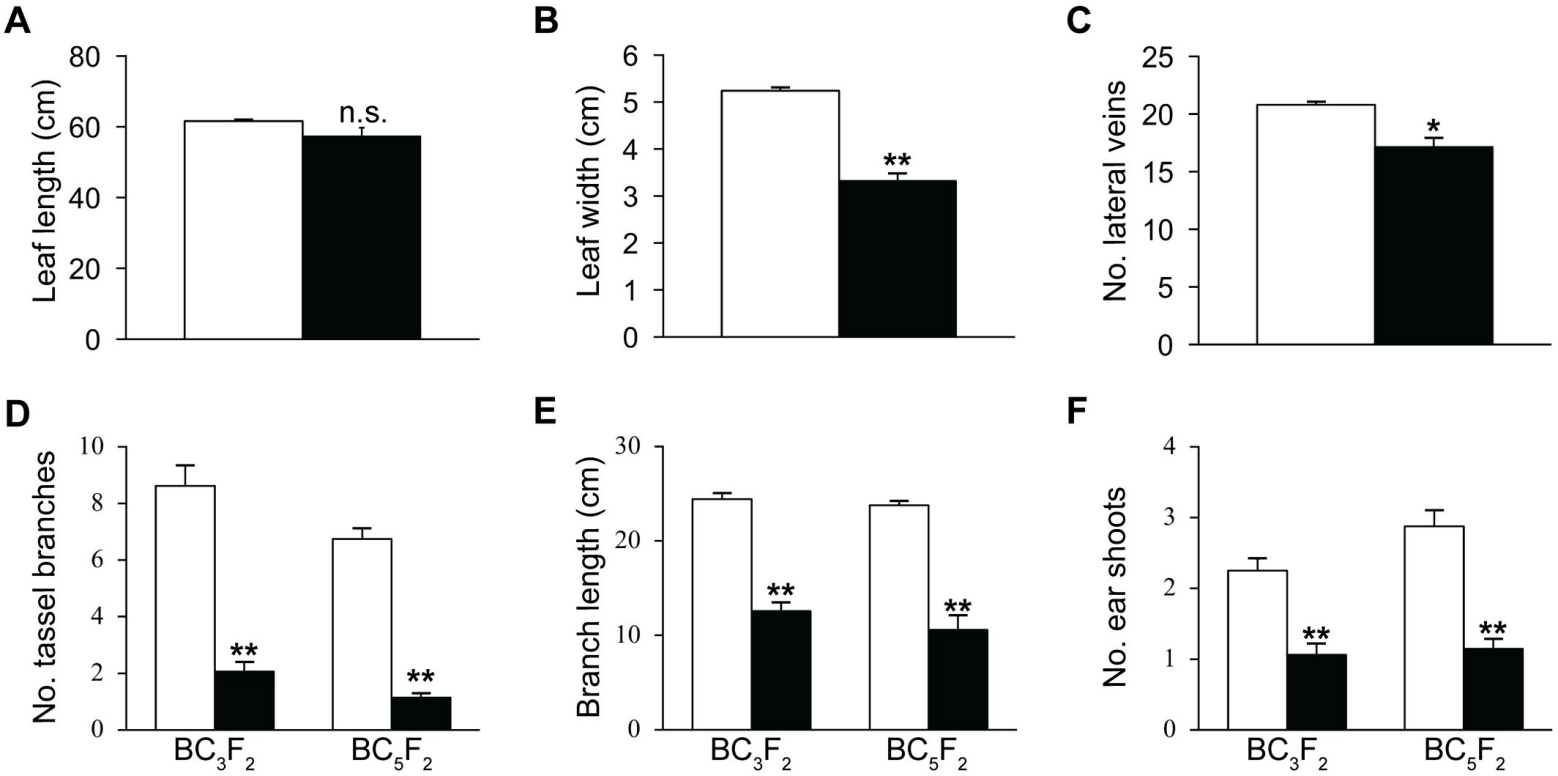
Morphometrics of *rmr12-3* mutant and non-mutant F_2_ siblings. Mean (±s.e.m.) of leaf 6 length **(A)** and width **(B)** of individuals from two progenies at flowering (mutant n = 38, non-mutant n = 236). **(C)** Mean number (±s.e.m.) of lateral veins at the midpoint of leaf 10 in 7-week-old plants from three progenies (mutants n = 6, non-mutants n = 10). **(D)** Mean tassel branch number (±s.e.m.) of individuals from eight progenies within the A632 background. BC_3_F_2_ (mutant n = 17, non-mutant n = 16). BC_5_F_2_ (mutant n = 7, non-mutant n = 8). **(E)** Mean primary tassel branch length (±s.e.m.) of individuals described in **(D)**. **(F)** Mean number of ear shoots (±s.e.m.) at flowering of individuals described in **(D)**. **(A)** to **(F)** solid bars: mutant, open bars: non-mutant. n.s. = not significant (*p*>0.05), * *p*<0.05, ** *p*<0.001.

Abaxial surfaces of *rpd1* mutant leaves can have adaxialized regions and occasionally exhibit ectopic outgrowths [12,13] but are generally indistinct from non-mutant leaves. In contrast, all *rmr12* mutant leaves are upright (Fig 1A and 1B) like *liguleless* mutants [67,68], adaxially-curled (Fig 1B) like Arabidopsis polycomb-group mutants [69], and have a textured/wrinkled epidermis (Fig 1C; see S1 Fig) similar to *crinkly4* mutants [70] and an RNAi knockdown of the maize BRASSINOSTEROID INSENSITIVE 1 ortholog (Zm-*bri1*) [71]. Leaf shape was also distinct. Among two independent progenies, mutant leaf 6 was no longer (*p* = 0.088, two-sample *t*-test), but clearly narrower (*p* = 5.07e-16, two-sample *t*-test), than the same leaf from non-mutant siblings (Fig 4A and 4B). Among three additional progenies evaluated, *rmr12* mutants had fewer lateral veins, (17.2 versus 20.8 respectively, *p* = 0.005, two-sample *t*-test) at the leaf 10 midpoint (Fig 4C).

*rpd1* mutant tassels are often feminized and always have a relatively compact architecture with short internodes between more acutely upright-angled secondary spikes [12] but male flowers extrude anthers that shed pollen normally. Although secondary spikes are similarly upright, *rmr12* mutant tassels rarely exhibit feminization, are severely reduced in both secondary spike numbers and primary spike lengths (1.8 vs 8 secondary spikes *p* = 2.07e-12, and 12 cm vs 24 cm primary spike length *p* = 9.5e-16, two-sample *t*-test for both) (Fig 4D and 4E), and the rarely-extruded anthers often fail to shed pollen. Manually extracted pollen appear visibly normal, however, and the progeny generated from mutant males indicates that at least some of their pollen is fertile (Fig 1E, Table 4; see S2 Table).

Cobs borne on *rpd1* mutants are smaller with heterogeneously sized kernels set in disorganized rows diagnostic of ovule abortions [12]. Similar, yet far more extreme, defects are seen in *rmr12* mutants with grain set being rare. Grain yields varied on mutant cobs with virtually none set in twelve successive Albany, CA summer nurseries compared with occasional sets on materials grown in Columbus, OH (Fig 1E). Fewer ear shoots were produced per plant (mean 1.1 vs. 2.5 ears *p* = 4.69e-9, two-sample *t*-test) in the eight F_2_ progenies examined (Fig 4F).

Overall, these *rmr12* mutant phenotypes are mostly distinct from those of *rpd1* mutants and instead mirror nearly all the known defects diagnostic of *pkl* loss-of-function mutants. As potential exceptions, some phenotypes including pickle-like root bulges on seedling roots and effects on light-dependent cotyledon opening [72,73] have not been adequately evaluated. Based on these apparent functional orthologies, molecular mapping data, sequence analyses, and phylogenetic relationships, we conclude that the *rmr12* locus encodes the maize PKL ortholog, hereafter referred to as CHD3a.

### CHD3a influences 24nt RNA biogenesis patterns

Because all known MOP and RMR proteins also maintain normal 24nt RNA levels [6,13,17,18,43–45,74], we compared PAGE-separated ethidium bromide-stained sRNAs isolated from sibling *rmr12-4 / rmr12-4* and *Rmr12 / rmr12-4* eight-day post-imbibition seedlings (see S7 Fig). Although similar comparisons clearly identify both RPD1- and RPD2a-dependencies [17], 24nt RNA levels appear undiminished in *rmr12-4* mutants.

**Fig 5:**
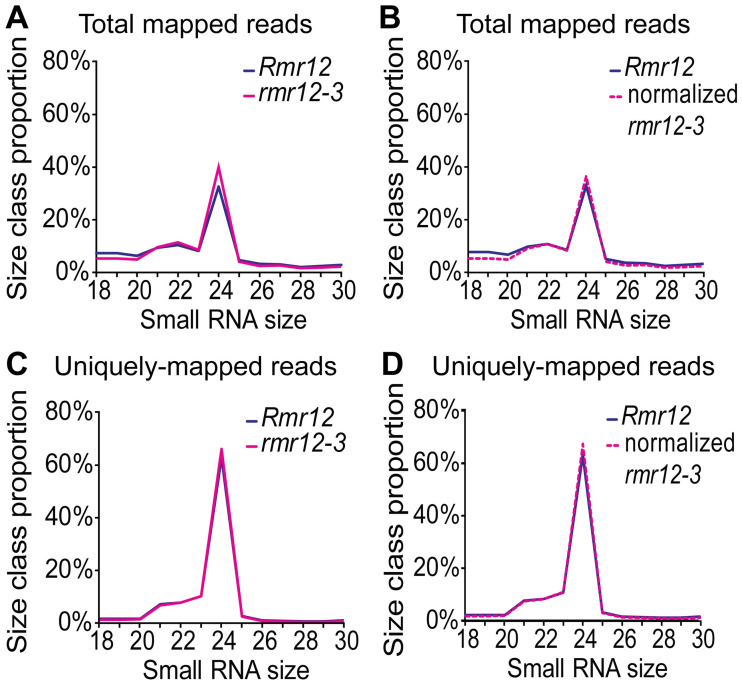
sRNA profiles in *rmr12-4* mutants. Size class distributions for all genome-mapped 18-30nt reads in *Rmr12 / Rmr12* and *rmr12-3 / rmr12-3* eight-day post imbibition seedlings **(A)** and normalized to 22nt RNA levels **(B)**. Distribution of uniquely-mapping 18-30nt reads **(C)** and normalized to 22nt RNA levels **(D)**.

To more precisely compare 24nt RNA profiles, we analyzed sRNA libraries from eight-day post imbibition seedlings homozygous for either *Rmr12* or *rmr12-3* (two non-mutant and three mutant libraries) by mapping the reads to the B73 reference genome AGPv4 [55] using ShortStack [75]. Comparing relative percentages of all mapped 18-30nt reads, we found no significant differences in 24nt read abundances between *rmr12-3* mutants and their non-mutant siblings (Fig 5A, *p* = 0.13, two-sample *t*-test). When percentages are normalized to the next most abundant size class (22nt), 24nt levels appear identical (Fig 5B). Additionally, both total and normalized 24nt read percentages are nearly identical for uniquely-mapped reads (Fig 5C and 5D) indicating CHD3a is not required for genome-wide 24nt RNA biogenesis. Of 59,127 sRNA genome clusters called on uniquely-mapped reads, 54,905 were predominantly 24nt. By DESeq2 analysis (see Methods), 14,656 (27%) of these 24nt clusters had differential read abundances of ≥2 fold change and *padj* (FDR) <0.05, with 7,848 increased and 6,808 decreased (see S6 Table), indicating that CHD3a influences where 24nt RNAs are produced.

Looking for possible *Pl1-Rhoades*-specific sRNAs, we aligned all 18-30nt reads which either mapped uniquely or did not map to the B73 genome to a 16kb lambda clone sequence containing the *Pl1-Rhoades* coding region (GenBank L19494) and upstream sequence using ShortStack. None of the seven clusters called across this sequence (see S7 Table; S8 Fig), had significant differences in normalized read counts (rpm) (see S8 Table). If CHD3a regulates *Pl1-Rhoades* through specific targeting of 24nt RNA production, this likely occurs 3´ of the existing *Pl1-Rhoades* haplotype sequence where previous recombination mapping identifies a *Pl1-Rhoades* enhancer and sequences conferring paramutagenic properties [7].

### CHD3a maintains repression of paramutant *Pl1-Rhoades*

**Fig 6:**
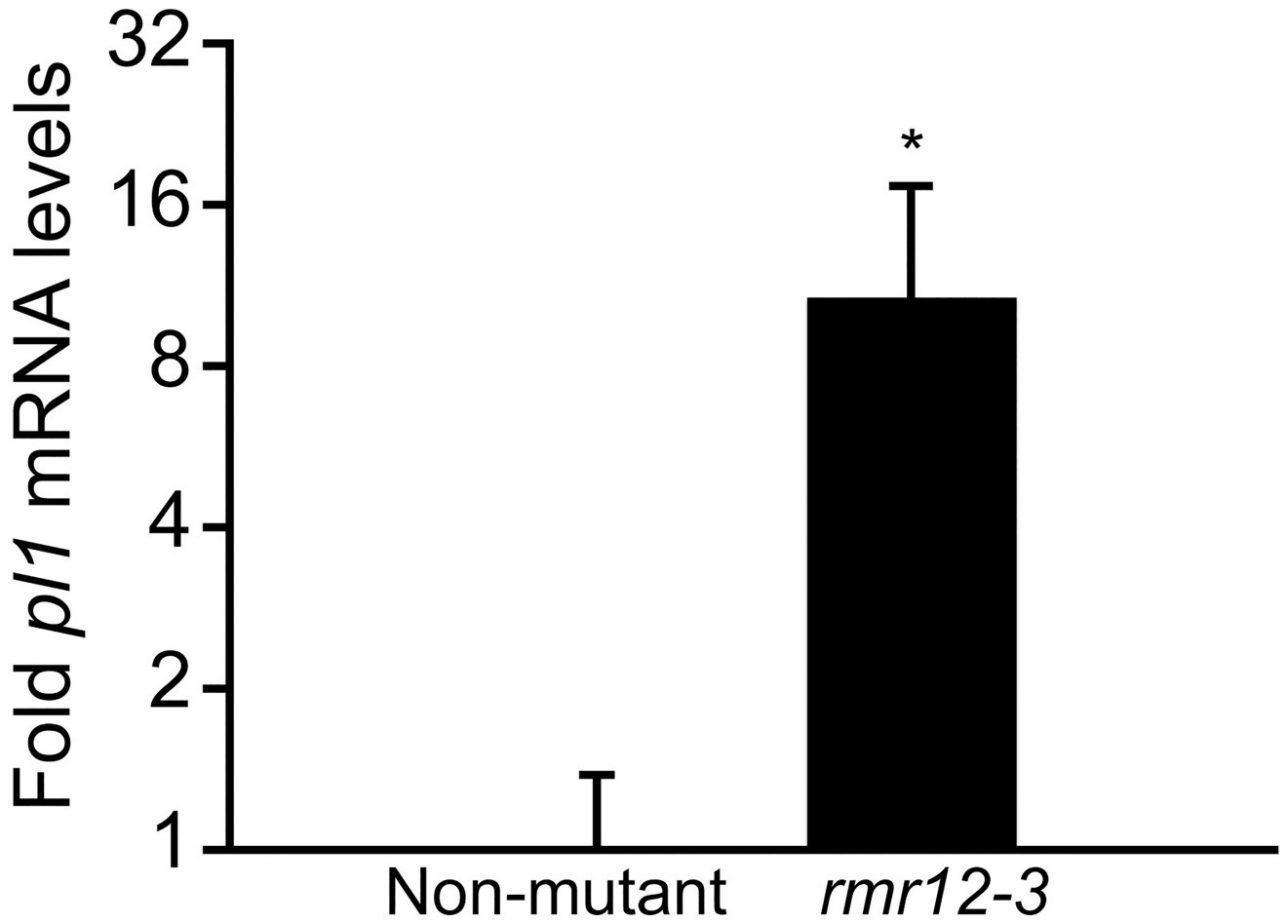
Relative *pl1* mRNA levels in *rmr12* mutants. Mean fold *pl1* mRNA expression (2^-ΔΔCt^) (±s.e.m) by qRT-PCR normalized to *gapdh* levels in biological triplicate non-mutant and *rmr12-3 / rmr12-3* eight-day post imbibition seedlings. * *p*<0.05.

The sRNA results led us to question whether the mutants’ increased pigmentation was specifically due to *Pl1-Rhoades* derepression or to a general increase in anthocyanin production independent of *pl1* function. To test this idea, we measured relative *Pl1-Rhoades* mRNA levels in *rmr12-3* mutant and non-mutant siblings by qRT-PCR and found on average 10 fold more *pl1* transcripts in *rmr12-3* mutants (Fig 6) supporting a role for CHD3a in maintaining either the transcriptional, post-transcriptional, or co-transcriptional repression of the *Pl´* state.

To address whether or not the elevation of *Pl1-Rhoades* mRNA was by itself sufficient to account for increased pigment, we synthesized mutants homozygous for functional yet recessive *pl1* alleles and evaluated their anther pigment phenotypes. Among the twenty F_2_ *rmr12-1* mutants segregating both *pl1-B73* and *Pl1-Rhoades* in a *Pl´* state, four had lightly-colored or near-colorless anthers typical of *pl1-B73* homozygotes, six had no florets, and ten displayed darkly pigmented anthers indistinguishable from that conferred by *Pl1-Rhoades* in a *Pl-Rh* state (Table 8). Similarly, among the ten F_2_ *rmr12-2* mutants segregating *Pl´* and *pl1-A632*, three had lightly-colored or near-colorless anthers (Table 8). The presence of anther phenotypes typical of recessive *pl1* expression in *rmr12* mutant individuals indicates that CHD3a does not generally enhance pigment production and confirms that increased anther pigmentation in *rmr12* mutants occurs from *Pl´* derepression.

**Table 8:** Anther phenotypes of F_2_ mutants segregating recessive *pl1* alleles.

Progeny				No. mutants with given anther phenotypes		
ID	Allele	*pl1* allele	No. plants	No anthers	Light/colorless	Pl-Rh-like
013120	*rmr12-1*	*pl1-B73*	104	6^a^	4	10
013115	*rmr12-2*	*pl1-A632*	60	2^b^	3	5

^a^Plants had either no or barren tassels, or insect damaged florets.

^b^One plant with a dry tassel and one plant with a vestigial tassel.

We next asked whether *Pl1-Rhoades* alleles could change from *Pl´* to meiotically heritable *Pl-Rh* states in the absence of CHD3a function. Once *Pl1-Rhoades* changes from *Pl-Rh* to *Pl´* it is always sexually transmitted in a *Pl´* state [23], even in the absence of some proteins required to maintain *Pl´* repression, including RMR2 [43] and RPD2a [17]. *Pl´* can, however, heritably revert to *Pl-Rh* at various frequencies when either hemizygous [76,77], heterozygous with specific recessive *pl1* alleles [76,77], or in *rmr1* and *rpd1* mutants [13,16,40,43]. To test if similar reversions occur in the absence of CHD3a, we crossed *Pl´ / Pl´ rmr12* mutants by five distinct *Pl-Rh* / *Pl-Rh* testers and then evaluated the progeny anther color phenotypes. If *Pl´* heritably reverts to *Pl-Rh* in the absence of CHD3a function, then some or all testcross progeny plants would have darkly pigmented anthers. Three of twelve progenies representing three distinct *Pl-Rh* / *Pl-Rh* testers had individuals (11 total) with intermediate or Pl-Rh-like anther colors (Table 9). These data, while relatively few in number, indicate that CHD3a contributes to maintaining meiotically-heritable information both specifying *Pl1-Rhoades* repression and facilitating paramutation in the subsequent generation. It remains to be evaluated whether CHD3a is also required to mediate *pl1* paramutation. This evaluation will require combining *Pl-Rh* and *Pl´* states in an *rmr12* mutant and independently tracking the paramutation-inducing properties of each transmitted allele.

**Table 9:** Testcross *rmr12* / *rmr12*; *Pl´* / *Pl´* X *Rmr12* / *Rmr12*; *Pl-Rh* / *Pl-Rh* progeny phenotypes.

Progeny			No. individuals with given anther color scores		
ID	Allele	*Pl-Rh* Tester	1–4 (Pl´)	5–6 (intermediate)	7 (Pl-Rh)
180506	*rmr12-4*	A619	6	0	0
180713	*rmr12-4*	A619	10	0	0
180787	*rmr12-4*	A619	6	0	**3**
172611	*rmr12-3*	A632 *T*	1	0	0
172608	*rmr12-3*	B73 *T*	2	0	0
172613	*rmr12-3* / *rmr12-4*	B73 *T*	56	0	0
172618	*rmr12-3*	B73 *T*	26	0	0
172620	*rmr12-3*	B73 *T*	3	0	0
180788	*rmr12-4*	K55	8	0	0
180789	*rmr12-4*	K55	6	**1**	0
180790	*rmr12-4*	K55	3	0	0
172609	*rmr12-3*	W23	14	0	0
172612	*rmr12-3*	W23	6	0	0
172614	*rmr12-3*	W23	3	0	0
172615	*rmr12-3*	W23	4	0	0
172619	*rmr12-3*	W23	1	0	0
160777	*rmr12-4*	W23	0	**7**^a^	0
160778	*rmr12-4*	W23	9	0	0
Total			188	8	3

*T*: (*Pl1-Rh* carried on a *T6-9* interchange).

^**a**^Individual tassels had anther color scores ranging from 3–5.

**Fig 7:**
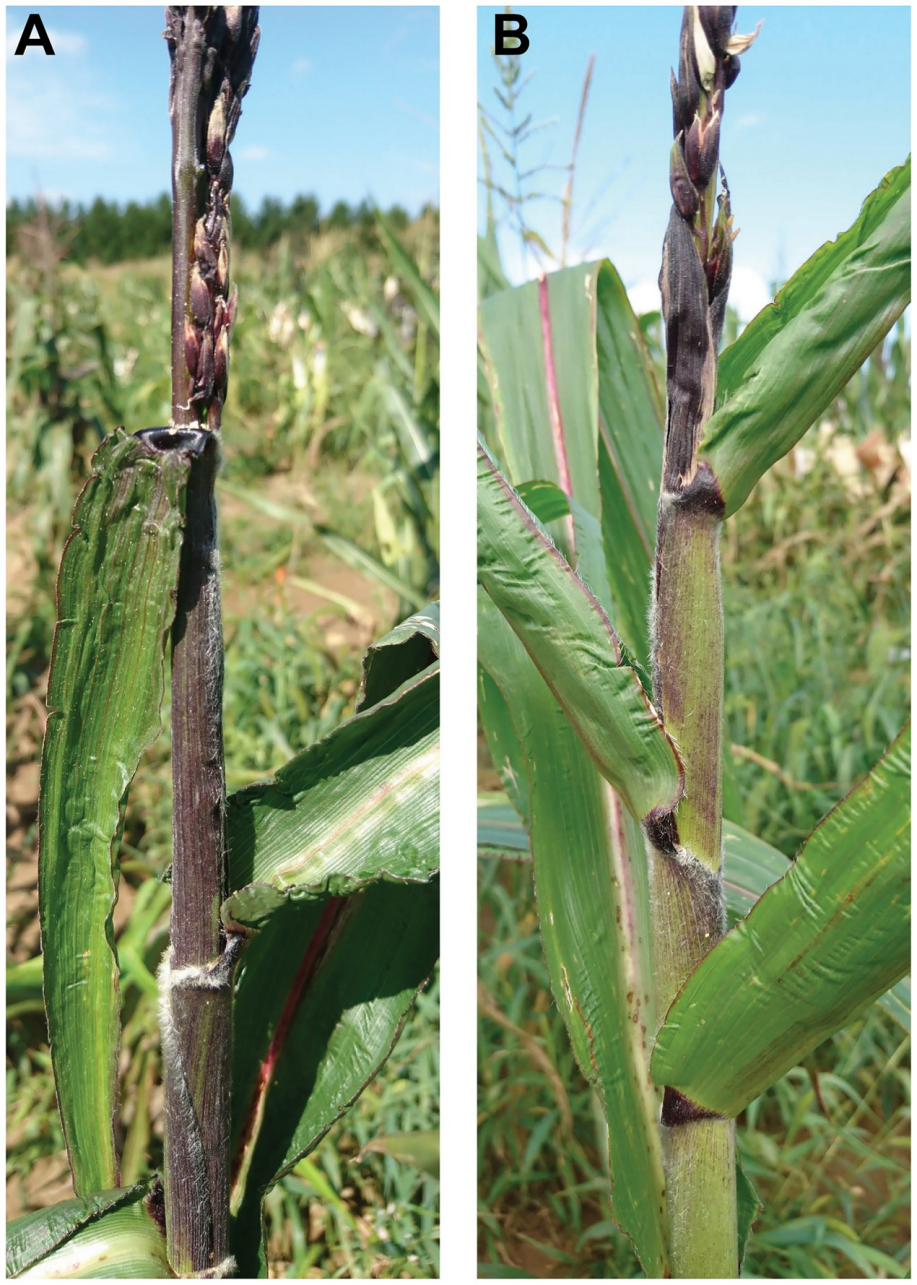
*rmr12* mutant *B1-I* phenotypes. *rmr12-4* mutants displaying *B-I* **(A)** and *B´* **(B)** states.

Because not all *rmr* mutations similarly affect paramutant alleles at other loci [17,43], we tested whether CHD3a also maintained repression of paramutant *b1* alleles by synthesizing *rmr12* mutants carrying *B1-I* alleles of either repressed (*B´*) or fully expressed (*B-I*) states (see S1 Methods). In both *mop1* and *rpd1* mutants, the *B´* state is derepressed such that it conditions dark leaf sheath pigmentation indistinguishable from that conferred by *B-I* [16,19,39]. Leaf sheath colors of the *B´* and *B-I rmr12* mutants were, in contrast, dissimilar (Fig 7) indicating that CHD3a is not required to maintain the *B´* state. To test if CHD3a is nonetheless required to mediate *b1* paramutation, *B´* / *B-I*; *rmr12-4* / *rmr12-4* individuals were synthesized (see S1 Methods), and testcrossed by recessive *b1* testers. All 26 individuals from three testcross progenies displayed a B´-like phenotype (Table 10) indicating that CHD3a does not mediate *b1* paramutation. We conclude that CHD3a function acts to maintain locus-specific repression at *Pl1-Rhoades*.

**Table 10:** Phenotypes of *rmr12-4 / rmr12-4; B´/ B-I* X *Rmr12 / Rmr12; b1 / b1* testcross progeny.

Progeny		No. Individuals having indicated phenotype	
ID	*b1* tester	B´-like	B-I-like
180787	A619	11	0
180788	K55	8	0
180789	K55	7	0
Total		26	0

## Discussion

CHD3a represents the first molecular component maintaining *Pl1-Rhoades* paramutations seemingly outside of an RdDM-type mechanism. Because CHD3a specifically maintains repression of *Pl1-Rhoades* but not *B1-I*, these results reaffirm that paramutation behaviors occurring at distinct loci can be mechanistically distinct [17,43]. Although the RPD1 and MOP1 requirement at multiple loci [16,39] support the involvement of an RdDM-like feed-forward loop reinforcing an RNAP II-repressive chromatin state [10], the initiation, and/or maintenance of such locus-specific regulatory loops might be differentially sensitive to the actions of other factors affecting allele-specific RNAP II transcriptional control.

Identification of *rmr12* as encoding a potential chromatin remodeling protein implicates the involvement of nucleosome alterations in maintaining paramutant states. In mammals, CHD3 proteins are complexed with HDACs and CpG- binding proteins [78] that facilitate transcriptional repression in hand with PRC2-mediated H3K27 methylation [79]. PKL, which might function as a monomer [80], also promotes H3K27me3 [81–84] and both positively and negatively affects mRNA levels of various H3K27me3-marked genes [81,82,84]. Despite these associations, it is unclear how PKL promotes H3K27me3 or whether it recognizes H3K27me3 *in vivo*, although the PHD domain from the rice CHD3, CHR729, has H3K27me3 affinity [60]. It is proposed that PKL promotes retention of H3K27me3-marked H2A.Z-containing nucleosomes through maturing prenucleosomes following transcription, an idea supported by the observation that PKL increases the size of DNase-resistant prenucleosome DNA fragments *in vitro* [84].

Based on the known biology of other CHD3 proteins and complexes [58,85], existing correlations between PKL function and H3K27me3 [81–84], and associations between mammalian CHD3 proteins, Histone Deacetylases (HDACs), and Polycomb Repressive Complex 2 (PRC2) [79], we envision a model in which CHD3a is required to maintain transcriptionally repressive (H3K27me3) nucleosomes that specify the *Pl´* state. Because CHD3 complexes can operate at transcriptional enhancers [85], one specific hypothesis is that CHD3a acts to continually repress RNAP II transcription at the *Pl1-Rhoades* 3´ enhancer—a feature co-mapping with sequences required for facilitating paramutation [7]—as a prerequisite for RNAP IV to compete for such templates [6,8]. It will be critical to identify these key regulatory sequences and evaluate their nucleosome, 24nt RNA, and nascent transcription profiles to test this idea.

The *Pl´* state is meiotically maintained by RNAP IV-dependent mechanisms [13,16], potentially involving 24nt RNAs and/or cytosine methylation. Because CHD3a always maintains the *Pl´* state in the soma but reverted *Pl-Rh* states are sometimes transmitted from *rmr12* mutants, it could be that other RNAP IV-dependent mechanisms deliver H3K27me3 marks to key regulatory sequences and maintain these at a certain level or location, but in the absence of CHD3a these H3K27me3 profiles are vulnerable to loss, resulting in occasional transmission of *Pl1-Rhoades* alleles that have reverted from *Pl´* to *Pl-Rh*. Transmission of derepressed *Pl1-Rhoades* alleles from *rmr12* mutants implies that a RNAP IV-dependent feature maintaining meiotic heritability must be, in part, stabilized by CHD3a function, and that feature must be capable of recruiting additional repression machinery including CHD3a in the next generation.

Although the roles of RPD1 and CHD3a in development are largely distinct, some shared mutant phenotypes, including juvenile to adult phase delays and dysregulation of *Pl1-Rhoades*, suggest certain alleles are co-dependent on CHD3a and RNAP IV actions. The large-scale changes in 24nt RNA distributions observed in *rmr12-3* mutants indicate that CHD3a has a role in specifying where RNAP IV is recruited or functions. A similar relationship may also exist in Arabidopsis as PKL was found in a genetic screen as required to repress a luciferase (LUC) transgene driven by the *RD29A* promoter in a cytosine demethylase mutant (*ros1*) background [86], a screen that also identified several RdDM components [87]. Loss of *pkl* also resulted in genome-wide changes in both 24nt RNA and 5-methylcytosine (5mC) profiles but approximately half of all differentially methylated regions were hypermethylated [86] indicating that RdDM still occurs but in different locations. As the *pRD29A-LUC* silencing behaviors appear to share paramutation-like features [88], it is possible that RNAP IV and CHD3a co-repression is diagnostic of some paramutant alleles.

Although two *C*. *elegans* CHD3s, CHD-3 and LET-418, are required for gamete viability [89], CHD3’s roles in gamete transmission were previously unknown. CHD3a is one of only a few known proteins which when disrupted lead to male transmission ratio distortions. We found no reports in Arabidopsis that PKL-deficient gametophytes are similarly affected so it is possible that grasses have co-opted CHD3a for controlling pollen-specific genes. Our *C1* transmission and *in vitro* germination results are inconsistent with problems in CHD3a-deficient pollen tube germination or growth and thus imply impairment of either stigma recognition and/or penetration, chemotaxis, or sperm cell delivery in *rmr12* mutant gametophytes. Future pollen RNA-seq comparisons may identify the critical CHD3a targets and reveal the nature of this gametophyte dysfunction.

Maize represents a new model for understanding the role(s) of CHD3 proteins and their potential complexes. Its large physical size and abundance of staged monoecious reproductive tissues should be especially useful for understanding their functions in plant development. Investigating how CHD3a coordinates developmental phase changes as well as the phenotypic variation specified by RNAP IV and meiotically-heritable paramutations should help identify regulatory sequences of morphological significance. These sequences could be selected from existing germplasms or engineered to potentially breed adaptive or desirable traits. Identifying the genomic features that recruit CHD3a is an obvious next step in further defining the paramutation mechanism(s) and its relationship to the orderly changes in allele states occurring during development [35].

## Materials and methods

### Genetic materials and stock syntheses

Genetic nomenclature follows guidelines established for *Zea mays* and has been previously described [16]. All stocks contain functional alleles for all factors required for anthocyanin production in the anthers unless otherwise indicated. Hand pollinations were used for all stock syntheses. The *rmr12* mutants were mostly used as the female parent because of their reticent anther phenotypes. The two reference alleles (ems98738 and ems98924) and two additional alleles (ems063095 and ems143190) were isolated from ems-treated pollen as previously described in [40] and [41], respectively. See S1 Methods for descriptions of specific stock syntheses. Additional pedigree information is available on request.

### Phenotyping

All quantitative phenotyping was assessed on materials grown in Columbus, OH summer nurseries with the exception of the *rmr12-1* and *rmr12-2* height and flowering time measurements which occurred in Albany, CA summer nurseries. Transition leaves marking the juvenile to adult phase change were visually assessed. The first leaf with adult characteristics typically has dull edges (conferred by juvenile phase-specific cuticular waxes) and a glossy V-shaped section in the center. Because the transition leaf was difficult to identify in *rmr12-3* mutant plants, we used toluidine blue O staining [90] to distinguish juvenile and adult waxes. Visual assignment of *Pl1-Rhoades* expression utilized a previously described anther color score [33] where scores 1–4 represent *Pl´* states, 5–6 represent intermediate types, and 7 represents the fully expressed *Pl-Rh* reference state.

### Pollen function

Pollen viability was assayed by fluorescein diacetate (FDA) stain as previously described [91]. Viable pollen was quantified from images taken under blue light five minutes after fresh pollen was mixed with FDA solution. *In vitro* pollen germination was carried out by plating fresh pollen on solid 1X pollen germination media [92] containing 10% sucrose, 0.0005% boric acid, 10mM calcium chloride, 0.05mM potassium phosphate, 6% polyethylene glycol 4000, and 0.3% noble agar. After germinating for 40 minutes at room temperature, pollen was stained with iodine potassium-iodide solution (0.1% iodine, 1% potassium iodine), imaged, and germination frequencies and pollen tube lengths of *wx1* (red-brown) and *Wx1* (blue) types were quantified using the image analysis software, Fiji [93]. Because iodine potassium-iodide staining can cause pollen tubes to burst, *Wx1* pollen germination frequencies are reported relative to the germination frequency of *wx1* types for each sample rather than as raw frequencies.

### Statistics

The individual values used to generate means and graphs are available in the minimal data set (see S1 File). In cases where observed categorical variables were compared to expected frequencies, *p* values are based on chi-squared tests, and the chi-square values are given. Significance for comparing quantitative variables was based on two-sample *t*-tests assuming unequal variance (see S2 File).

### Recombination mapping and candidate gene analysis

A set of *rmr12-3* BC_3_F_2_ mutants was interrogated with molecular markers (see S9 Table for primer sequences and diagnostic enzymes) distinguishing parental A619 and A632 polymorphisms including simple satellite repeats (SSR) from the University of Missouri-Columbia (UMC) collection, newly designed cleaved amplified polymorphic sequences (CAPS), and derived CAPS (dCAPS), and the frequency of A619 alleles was recorded (Table 7). Results of individual mutants tested with each marker indicated single recombination events between *rmr12-3* and both 9_12.38 and 9_16.47 in opposite directions indicating the *rmr12* locus was between these markers. A dCAPS marker identified the *rmr12-3* mutation, and *rmr12-4* was genotyped with a CAPS marker (see S9 Table).

The composite sequences from PCR amplicons of cDNA from each *rmr12* allele (see S5 Fig) and translated proteins were aligned to Zm00001d045109_T004 and P004, respectively, using the Geneious alignment tool ([94]; version 6.1.8) with mRNA, CDS, and protein domains predicted by simple modular architecture research tool (SMART) [95] (see S5 and S6 Figs). GenBank accessions for *rmr12-A619*, *rmr12-1*, *rmr12-2*, *rmr12-3*, and *rmr12-4* complete coding sequences are MK875675-MK875679.

### sRNA analysis

Low molecular weight RNA isolated from pooled eight-day post imbibition seedlings using TRIzol reagent (Invitrogen) and purified by chloroform and 5:1 acid phenol:chloroform extractions was enriched from total RNA by precipitating the majority of the high molecular weight RNAs by centrifugation (13,200 rpm for 10 minutes at 4C) in 11.5% PEG-8000, 38% formamide, 58mM NaCl. Low molecular weight RNAs were separated on a polyacrylamide gel (15% acrylamide/bis-acrylamide (19,1), 8M urea, 22.5mM Tris, 22.5 mM boric acid, 0.5mM EDTA) and stained with ethidium bromide.

sRNA libraries were made from total RNA isolated from individual eight-day post imbibition F_2_ sibling seedlings homozygous for either *Rmr12* or *rmr12-3* using TRIzol reagent (Invitrogen) according to the manufacturer’s instructions. Libraries were prepared using the gel-free size selection method of the BIOO NEXTFLEX Small RNA-Seq Kit v3 (Perkin Elmer). A pool of three mutant and non-mutant single indexed libraries was sequenced (150bp paired-end) on a single HiSeq 4000 lane by Novogene Co. Ltd. One of the non-mutant replicates produced few reads (less than 20,000) and was excluded from further analysis. The *BBTools* (https://jgi.doe.gov/data-and-tools/bbtools/) function *bbduk* was used to trim adapter sequences, remove low-quality reads, and retain 18-30nt reads with options (ktrim=r k=18 mink=11 hdist=1 tp=4 ftl=4 minlen=18 maxlength=30). The combined five libraries produced 182 million high-quality sRNA read pairs (see S10 Table). Mate 1 representing sense reads from each library was aligned to the B73 reference genome AGPv4 [55] not allowing mismatches or multiply-mapping reads, and clusters were called using ShortStack [75] with options (--mismatches 0 --mmap n --nohp --pad 75 --mincov 91). Because the ShortStack default settings for these datasets would allow a cluster to have as few as 3.4 reads per library, we set a non-default minimum coverage of 91 reads across all libraries (representing 0.5 rpm) to avoid differential cluster calls on regions with questionable biological significance. Counts from clusters defined as primarily 24nt by ShortStack were compared using DESeq2 [96], and those with log_2_ fold change ≥1 and *padj* (FDR) <0.05 were considered significant. These data are available through GEO (GSE158990).

To investigate sRNA clusters near *Pl1-Rhoades*, sRNA reads were first aligned to the B73 reference genome AGPv4 [55] using Bowtie (v0.12.8; options: -v 0 --best -m 1 -S) [97]. Multiply-mapping reads were excluded to ensure mapped sRNAs derived only from *Pl1-Rhoades* proximal sequences. Reads remaining unmapped or that mapped uniquely were subsequently aligned and clustered to the recently updated *Pl1-Rhoades* sequence from GenBank L19494 using ShortStack with the above parameters. The original L19494 sequence, representing the coding region and limited flanking sequences of a *Pl1-Rhoades*-containing lambda clone [98] was extended to include 11.6kb additional 5´ sequence with no gaps by sequencing lambda subclones and supplementing with a short genomic PCR amplicon as described [99,100]. Read counts for all seven clusters were converted to rpm 18-30nt clean reads, and differential expression was tested with 2-sample *t*-tests (see S8 Table).

### qRT-PCR analysis

Total RNA was isolated from the first and second leaf sheaths below the lowest leaf blade of fourteen-day post imbibition *Rmr12* and *rmr12-3* homozygous F_2_ siblings using TRizol reagent (Invitrogen) as specified by the manufacturer. Tissues from two seedlings were pooled per sample. Isolated RNA was treated with DNase I (Roche), and 500ng of RNA was reverse transcribed using Protoscript II (NEB) and oligo(dT) primers. The resulting cDNA was treated with RNase A/T1, and one twenty-fifth of the RT reaction was included in technical triplicate 20μl PCR reactions with SensiMix SYBR No ROX (Bioline). Data were generated using an Eppendorf Mastercycler EP Gradient S thermocycler, and cycle threshold (Ct) values were calculated using the noiseband option in Eppendorf Mastercycler EP Realplex V2.2 software. *pl1* transcripts were amplified with primers recognizing exon 1 (see S9 Table) and normalized to *gapdh* levels amplified using previously published primers [101] (see S9 Table).

### Phylogenetic analysis

The SMART-predicted DUF1087 amino acid sequence from Zm00001d045109_P008 was used as the BLASTp query for *A*. *thaliana* and the grasses included in Phytozome v12.1 (https://phytozome.jgi.doe.gov/pz/portal.html) [102] except that maize sequences were replaced with those from B73 AGPv4 [55] obtained from Gramene (http://ensembl.gramene.org/Zea_mays/Info/Index). For each protein match from B73 AGPv4, the predicted isoform encoding the longest amino acid sequence was included for analysis. Alternative isoforms were removed from Phytozome v12 matches, and Oropetium_20150105_13389 was also removed because it lacked all other CHD domains. The full-length amino acid sequences from all protein matches, and *S*. *cerevisiae* CHD1 from Uniprot, were aligned using the MUSCLE alignment tool in Geneious ([94]; version 6.1.8) (see S3 File), and a maximum likelihood tree was created with Phyml [103] using the JTT amino acid substitution model and NNI+SPR tree topology search operation with 1000 bootstrap iterations (see S4 File). The resulting tree was oriented to display *S*. *cerevisiae* CHD1 (a founding member of the CHD clade of SNF2-ATPases) as the root using Geneious ([94]; version 6.1.8).

## Supporting information

S1 Fig Mutant plant phenotypes. Additional mutant leaf blade phenotypes in ems063095 **(A)** and ems143190 **(B)** mutants.
(TIFF)

S2 Fig Cob phenotypes. **(A)** Cob from self-pollination of an *Rmr12 c1* / *rmr12-4 C1* individual. **(B)** *c1* / *c1* X *Rmr12 c1* / *rmr12-4 C1* test cross cob holds progeny 170323.
(TIF)

S3 Fig Pollen phenotypes. **(A)** Frequency of viable pollen (stained with fluorescein diacetate) from four florets each from *Rmr12* / *Rmr12* and *Rmr12* / *rmr12-4* individuals. **(B)** Ratio of *Wx1* to *wx1* pollen germination frequencies from eight florets from a *Rmr12 wx1* / *rmr12-4 Wx1* individual. **(C)** *wx1* and *Wx1* pollen tube lengths (mm) from eight florets from a *Rmr12 wx1* / *rmr12-4 Wx1* individual. Boxplot whiskers encompass the range of data not including outliers (grey dots) which fall more than 1.5 X (interquartile range) above or below the box.
(TIF)

S4 Fig Intron retention in *rmr12-3* cDNAs. **(A)** Schematic representation of exons 22 and 23 in Zm00001d045109_T004 with the placement of primers (arrows) used to amplify B73 gDNA and cDNAs from *Rmr12* / *Rmr12* and *rmr12-3* / *rmr12-3* individuals **(B)**. Hatched box represents intronic sequence retained in *rmr12-3* mutants.
(TIF)

S5 Fig Alignments of Zm00001d045109 cDNA sequences. Partial mutant mRNA compiled from Sanger sequenced *Rmr12-A619* and mutant cDNA amplicons aligned to a predicted reference transcript, Zm00001d045109_T004. Red = mRNA, yellow = coding sequence.
(DOCX)

S6 Fig Alignments of Zm00001d045109 protein sequences. *rmr12* allele translations aligned to the Zm00001d045109_P004 reference protein sequence, with domains predicted by Simple Modular Architecture Research Tool (SMART).
(DOCX)

S7 Fig Bulk sRNA profiles in *rmr12-4* mutants. Ethidium bromide stained PAGE fractionated sRNAs from pooled *Rmr12* / *rmr12-4* or *rmr12-4* / *rmr12-4* eight-day post-imbibition seedlings. Sizes in nucleotides (nt) are shown.
(TIF)

S8 Fig sRNA alignments to the *Pl1-Rhoades* region. **(A)** Uniquely-mapping sRNA reads from each library aligned to a lambda clone sequence containing the *Pl1-Rhoades* coding region. Peak heights are scaled to library depth. **(B)** Clusters called by ShortStack with the relative position of the *Pl1-Rhoades* coding region **(C)**.
(TIF)

S1 Table Genetic complementation tests based on anther pigments. (DOCX)

S2 Table Genetic complementation tests based on developmental defects. (DOCX)

S3 Table F_2_ *rmr12* mutant frequencies. (DOCX)

S4 Table *rmr12-1* and *wx1* cosegregation in progenies from self-pollinated *Rmr12* / *rmr12-1*; *Wx1* / *wx1* plants. (DOCX)

S5 Table Gene models within the *rmr12* mapping interval. (XLSX)

S6 Table *rmr12-3* differentially represented sRNA clusters. (XLSX)

S7 Table *Pl1-Rhoades* cluster characteristics. (XLSX)

S8 Table *Pl1-Rhoades* proximal sRNA cluster representation in *rmr12* mutants. (DOCX)

S9 Table Primers used in this study. (DOCX)

S10 Table Library statistics. (DOCX)

S1 Methods (DOCX)

S1 File Minimal data set. (XLSX)

S2 File Statistics. (DOCX)

S3 File CHD3 phylogram alignments. (TXT)

S4 File CHD3 phylogenetic tree. (TXT)
