# Supplementary figures and images for: Locus-specific paramutation in Zea mays is maintained by a PICKLE-like chromodomain helicase DNA-binding 3 protein controlling development and male gametophyte function
Source: PLoS Genet. 2020 Dec 15;16(12):e1009243. doi: 10.1371/journal.pgen.1009243 (PMC7837471; doi:10.1371/journal.pgen.1009243)

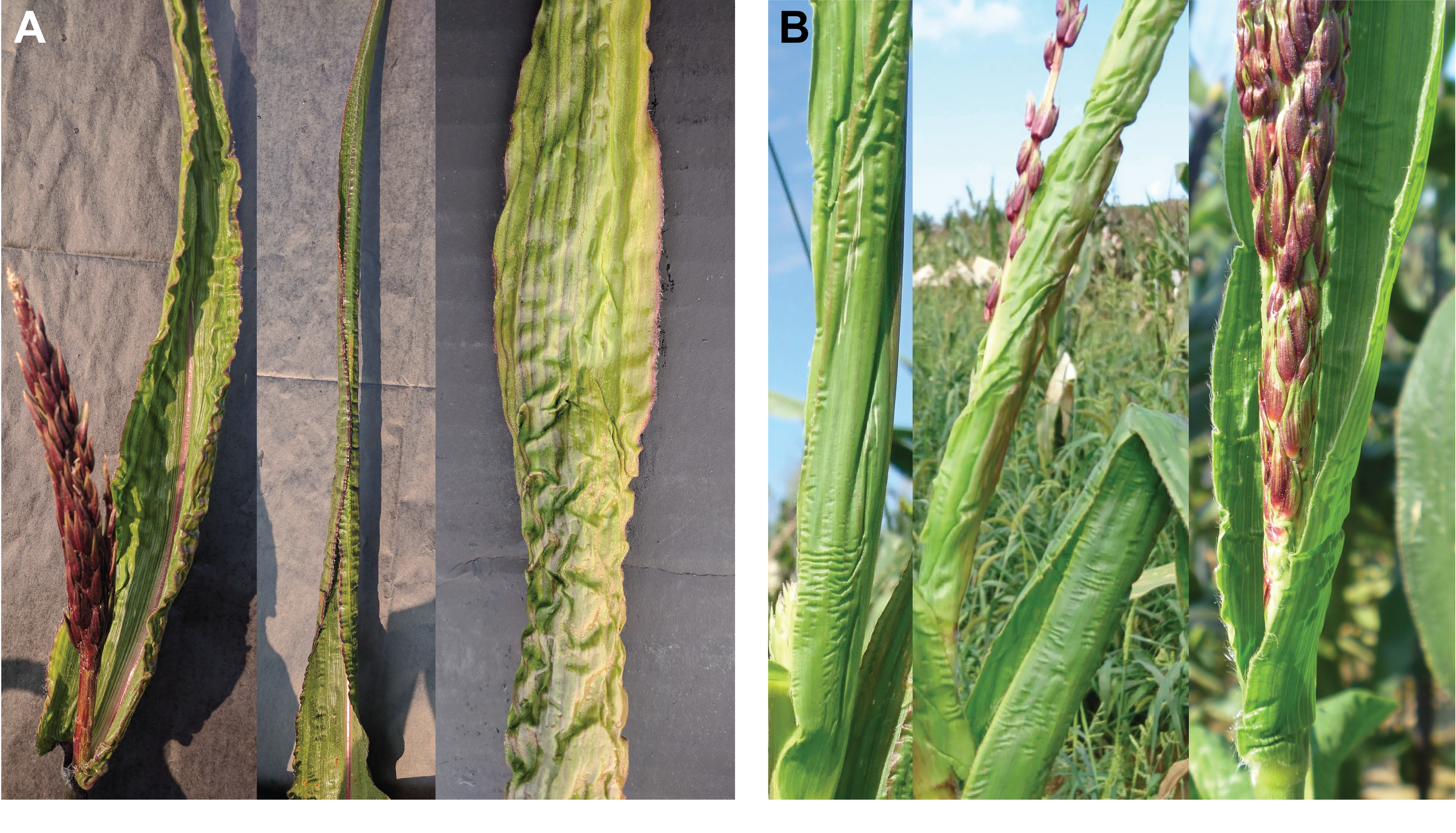

Supplement: S1 Fig — Additional mutant leaf blade phenotypes in ems063095 (A) and ems143190 (B) mutants. (TIFF) [file pgen.1009243.s001.tiff]

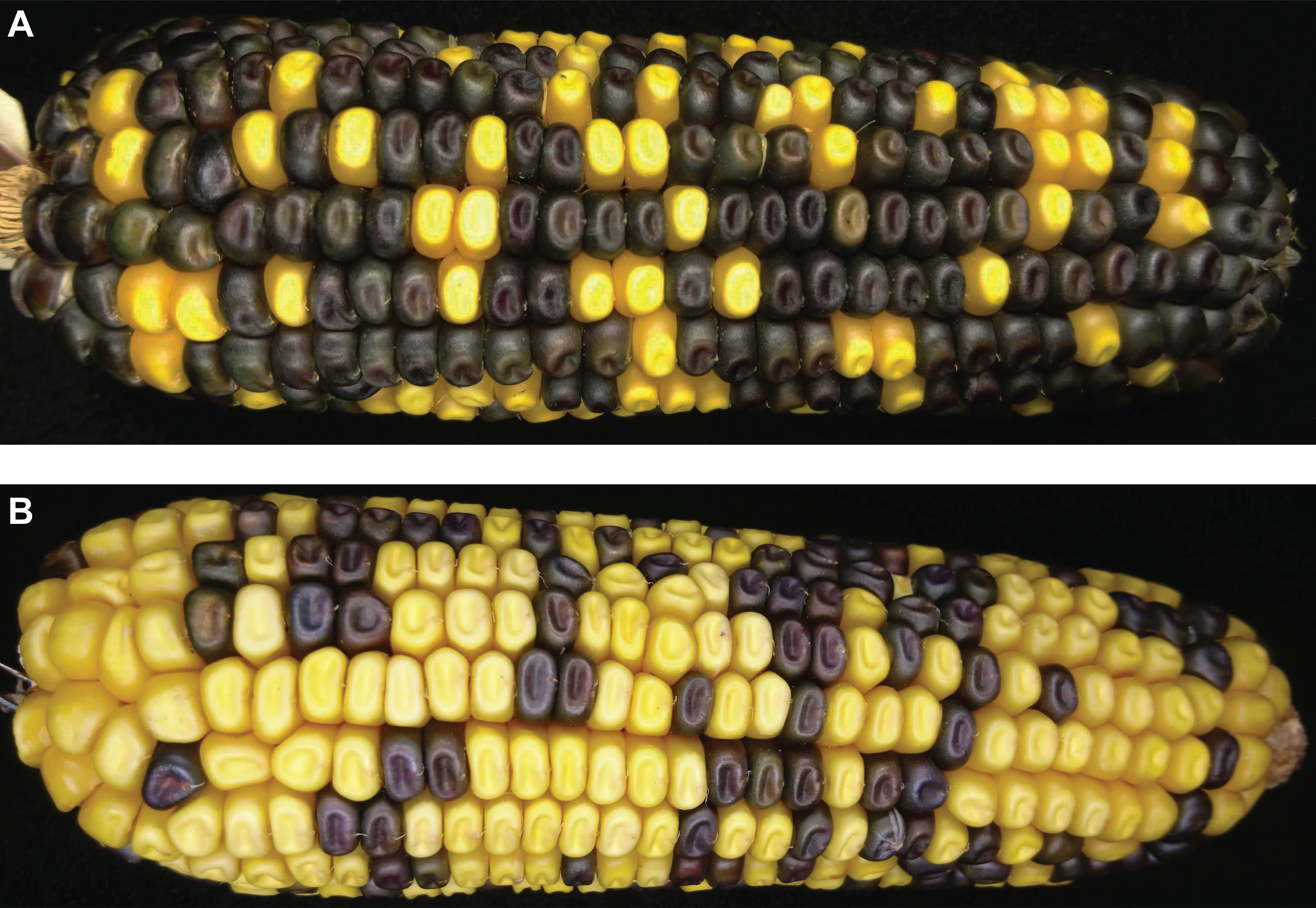

Supplement: S2 Fig — (A) Cob from self-pollination of an Rmr12 c1 / rmr12-4 C1 individual. (B) c1 / c1 X Rmr12 c1 / rmr12-4 C1 test cross cob holds progeny 170323. (TIF) [file pgen.1009243.s002.tif]

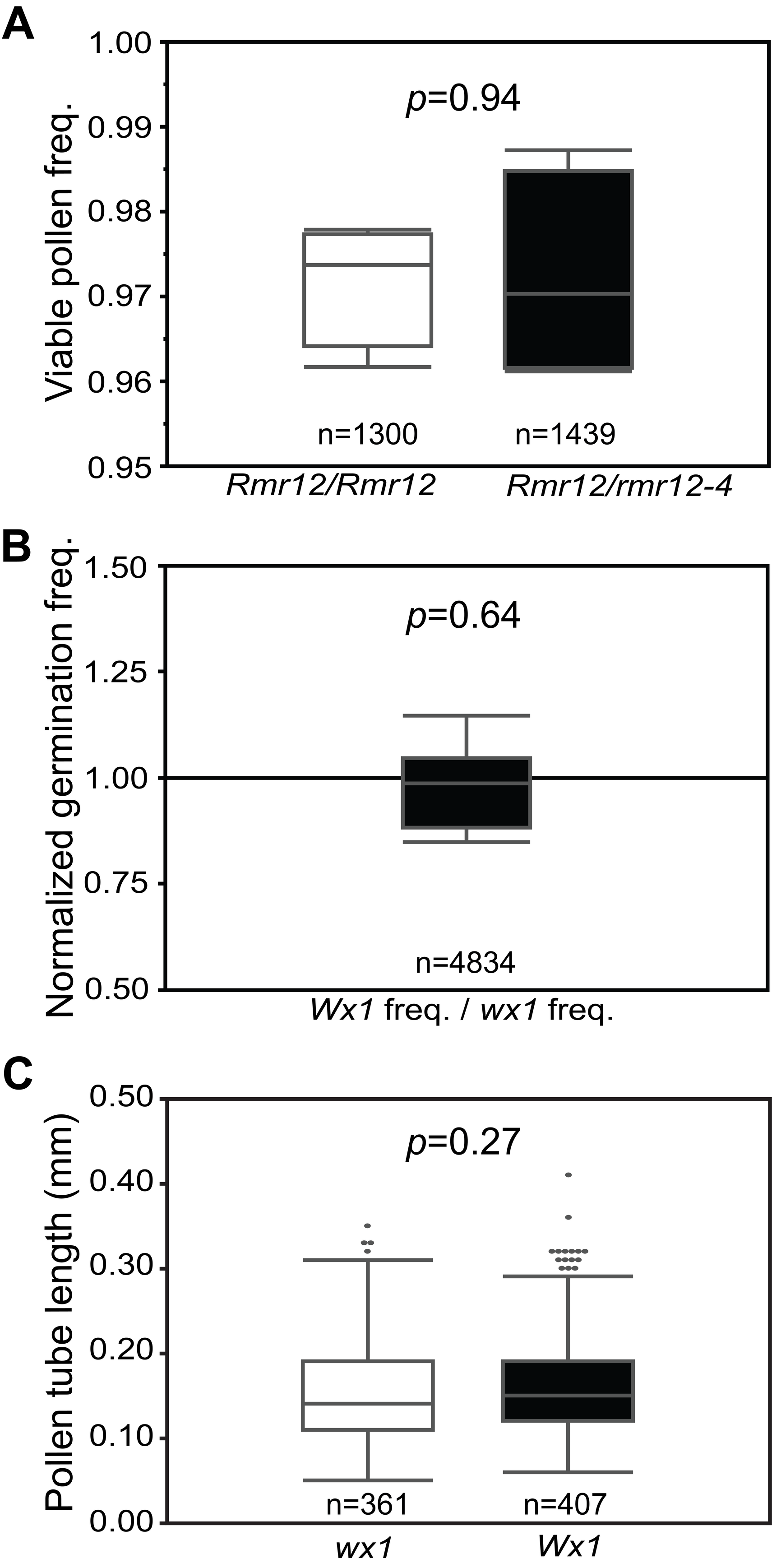

Supplement: S3 Fig — (A) Frequency of viable pollen (stained with fluorescein diacetate) from four florets each from Rmr12 / Rmr12 and Rmr12 / rmr12-4 individuals. (B) Ratio of Wx1 to wx1 pollen germination frequencies from eight florets from a Rmr12 wx1 / rmr12-4 Wx1 individual. (C) wx1 and Wx1 pollen tube lengths (mm) from eight florets from a Rmr12 wx1 / rmr12-4 Wx1 individual. Boxplot whiskers encompass the range of data not including outliers (grey dots) which fall more than 1.5 X (interquartile range) above or below the box. (TIF) [file pgen.1009243.s003.tif]

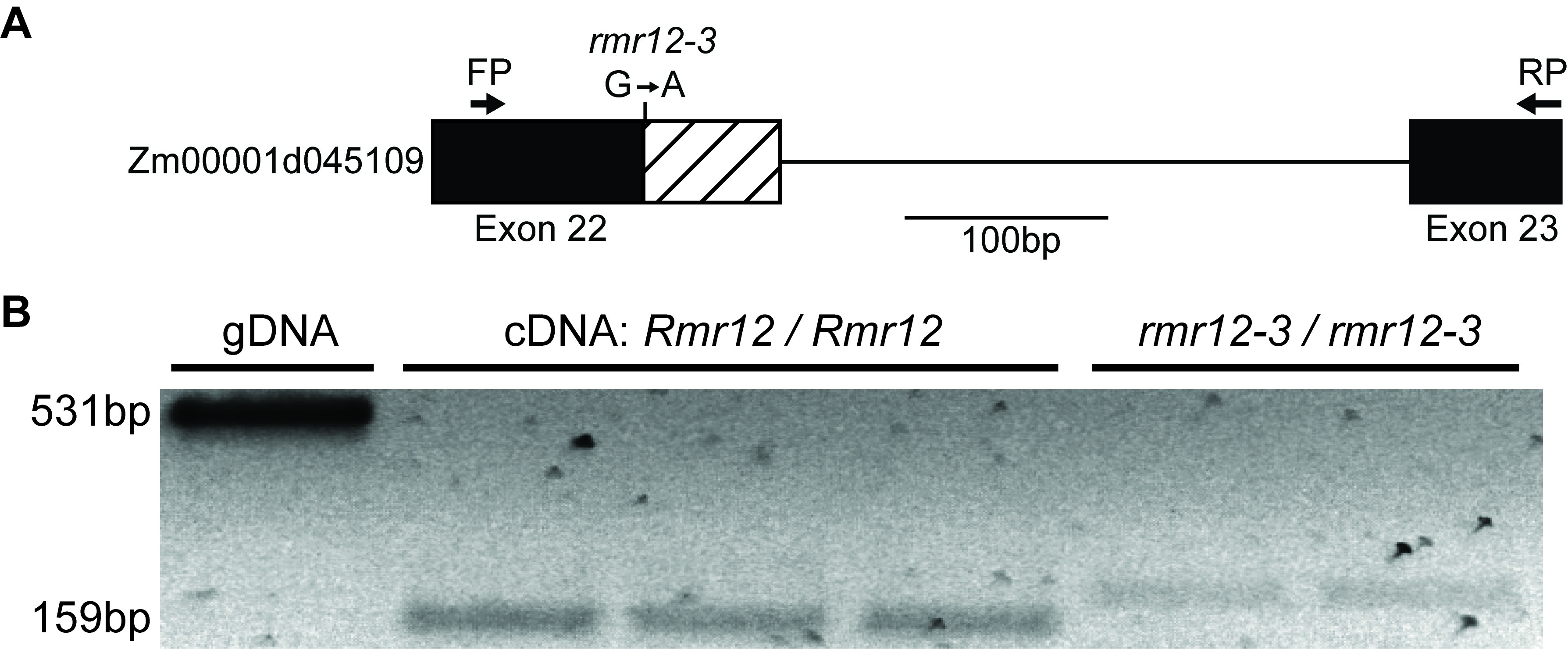

Supplement: S4 Fig — (A) Schematic representation of exons 22 and 23 in Zm00001d045109_T004 with the placement of primers (arrows) used to amplify B73 gDNA and cDNAs from Rmr12 / Rmr12 and rmr12-3 / rmr12-3 individuals (B). Hatched box represents intronic sequence retained in rmr12-3 mutants. (TIF) [file pgen.1009243.s004.tif]

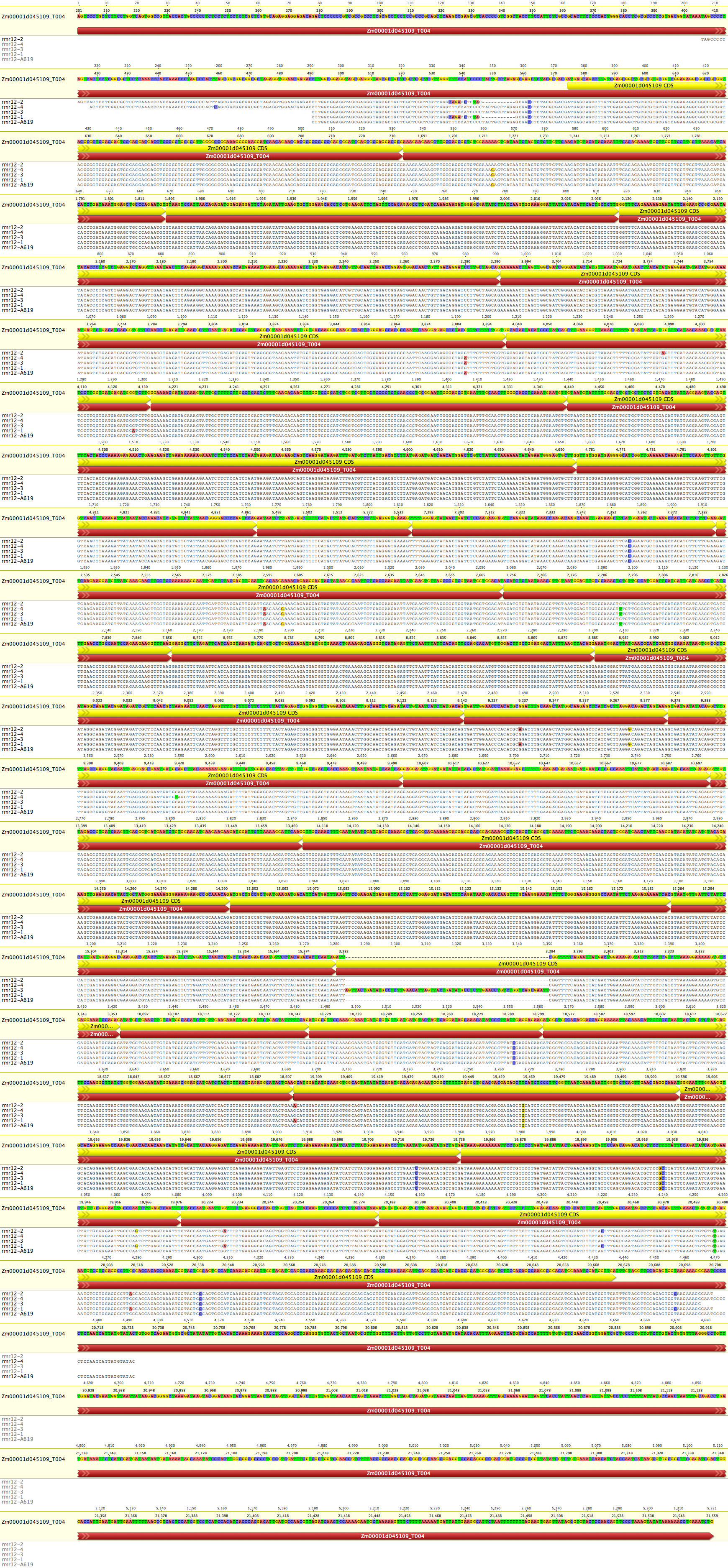


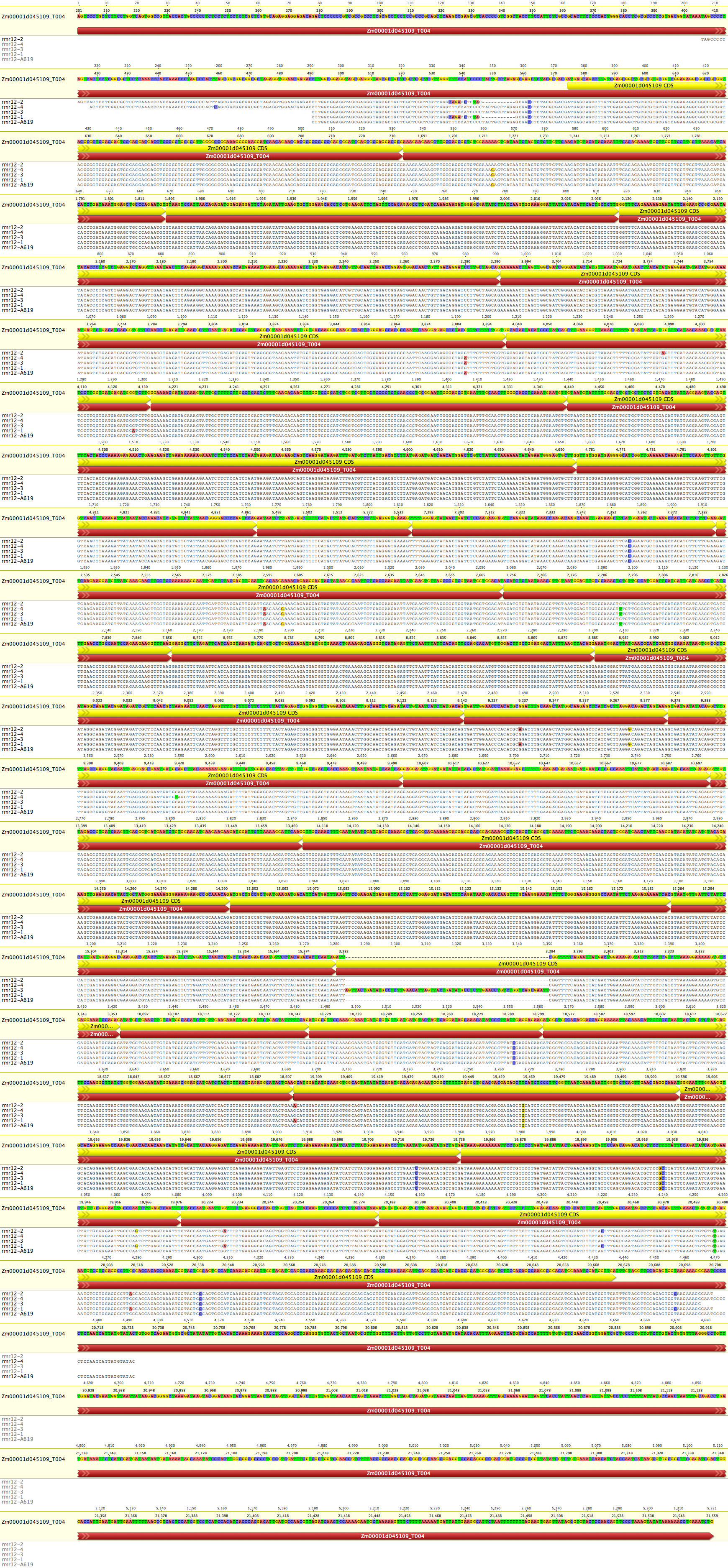


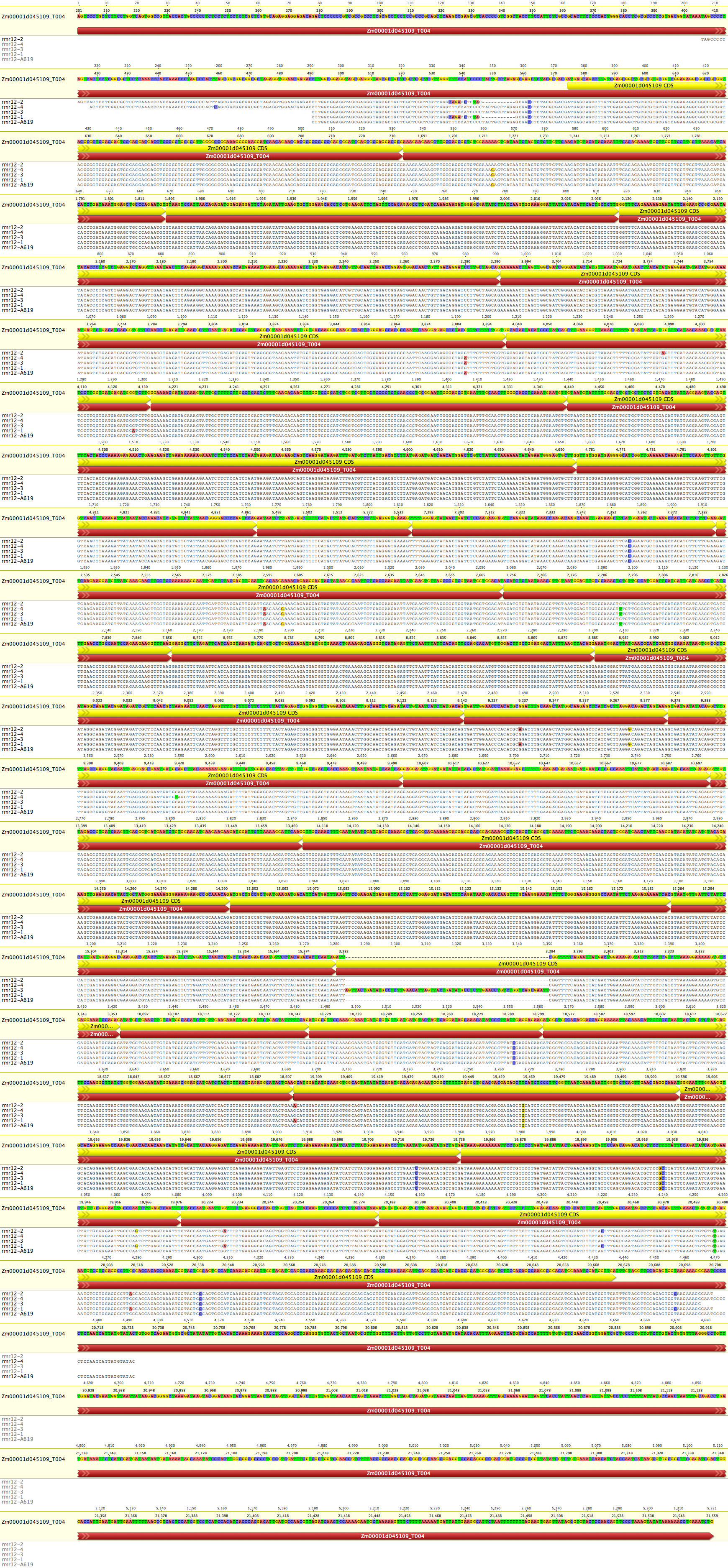

Supplement: S5 Fig — Partial mutant mRNA compiled from Sanger sequenced Rmr12-A619 and mutant cDNA amplicons aligned to a predicted reference transcript, Zm00001d045109_T004. Red = mRNA, yellow = coding sequence. (DOCX) [file pgen.1009243.s005.docx]

**
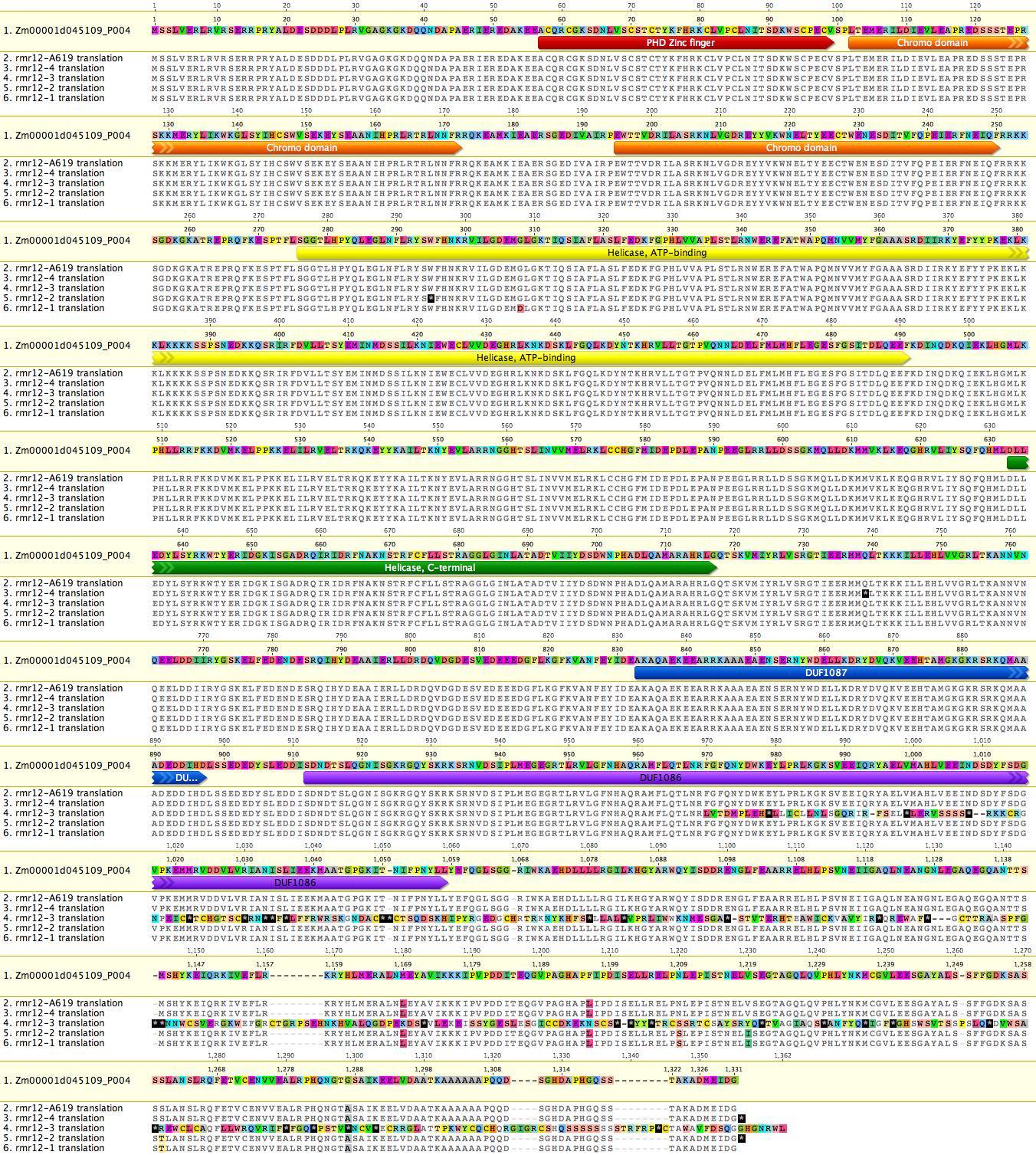
**

**
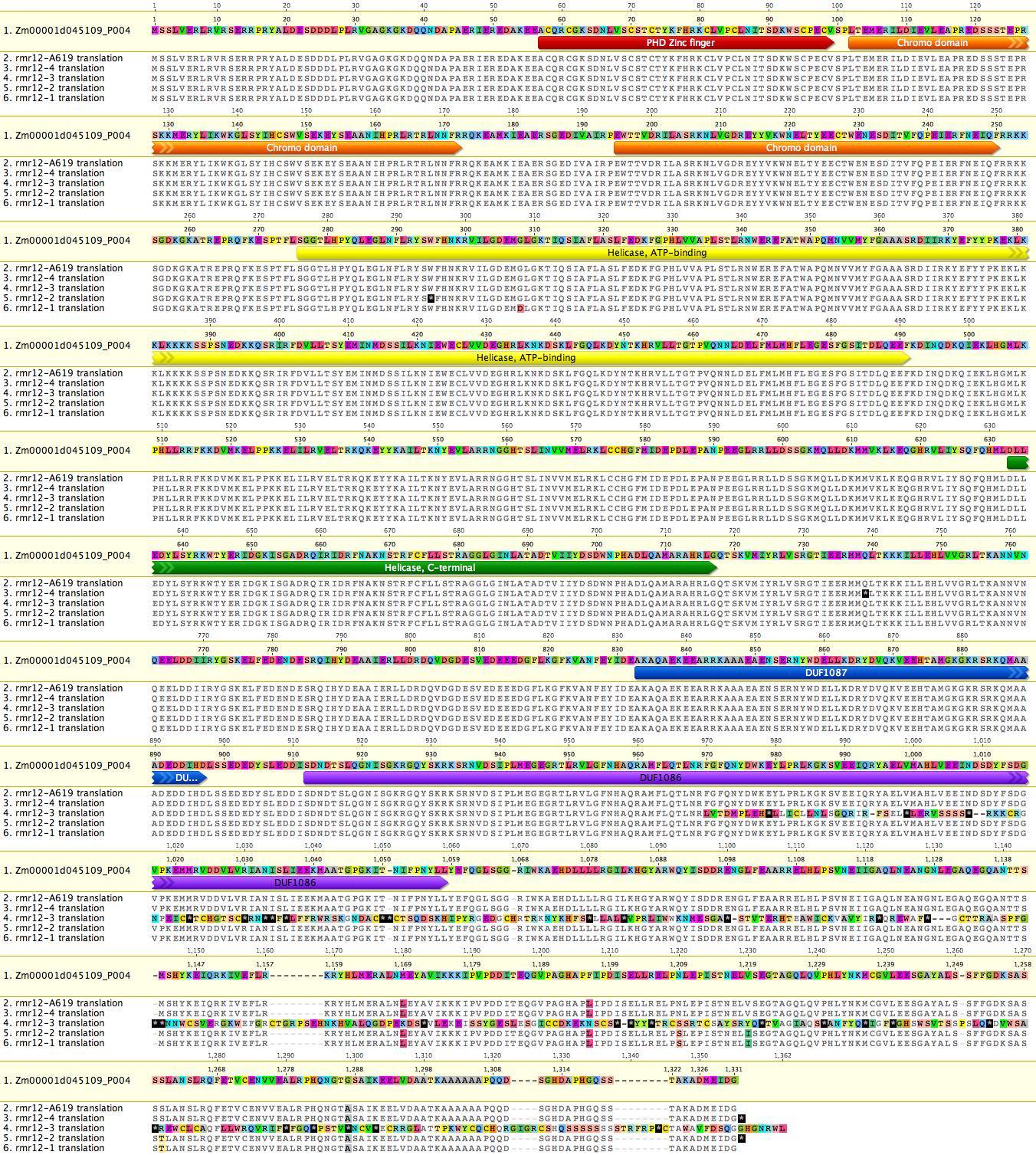
**

Supplement: S6 Fig — rmr12 allele translations aligned to the Zm00001d045109_P004 reference protein sequence, with domains predicted by Simple Modular Architecture Research Tool (SMART). (DOCX) [file pgen.1009243.s006.docx]

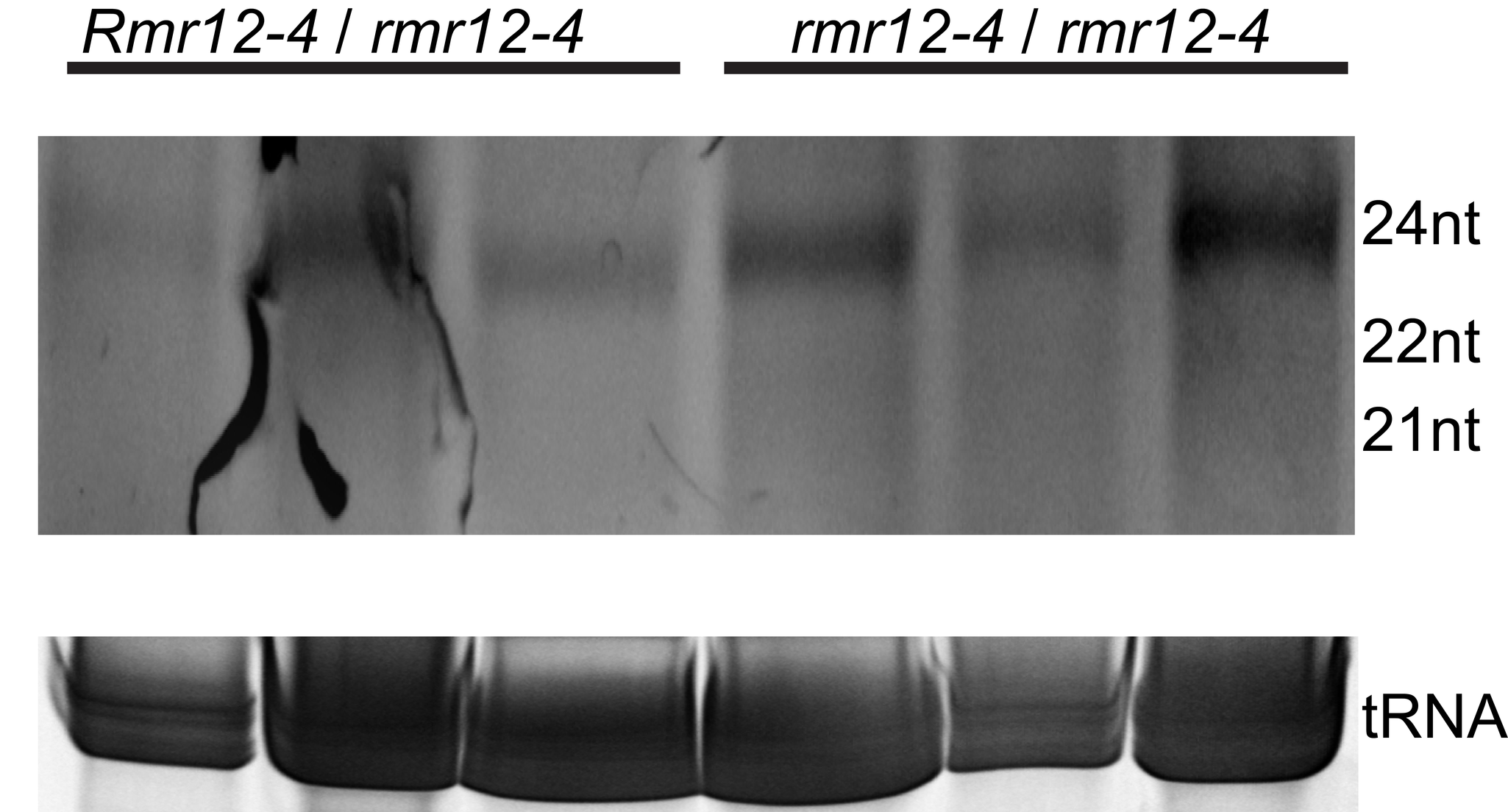

Supplement: S7 Fig — Ethidium bromide stained PAGE fractionated sRNAs from pooled Rmr12 / rmr12-4 or rmr12-4 / rmr12-4 eight-day post-imbibition seedlings. Sizes in nucleotides (nt) are shown. (TIF) [file pgen.1009243.s007.tif]

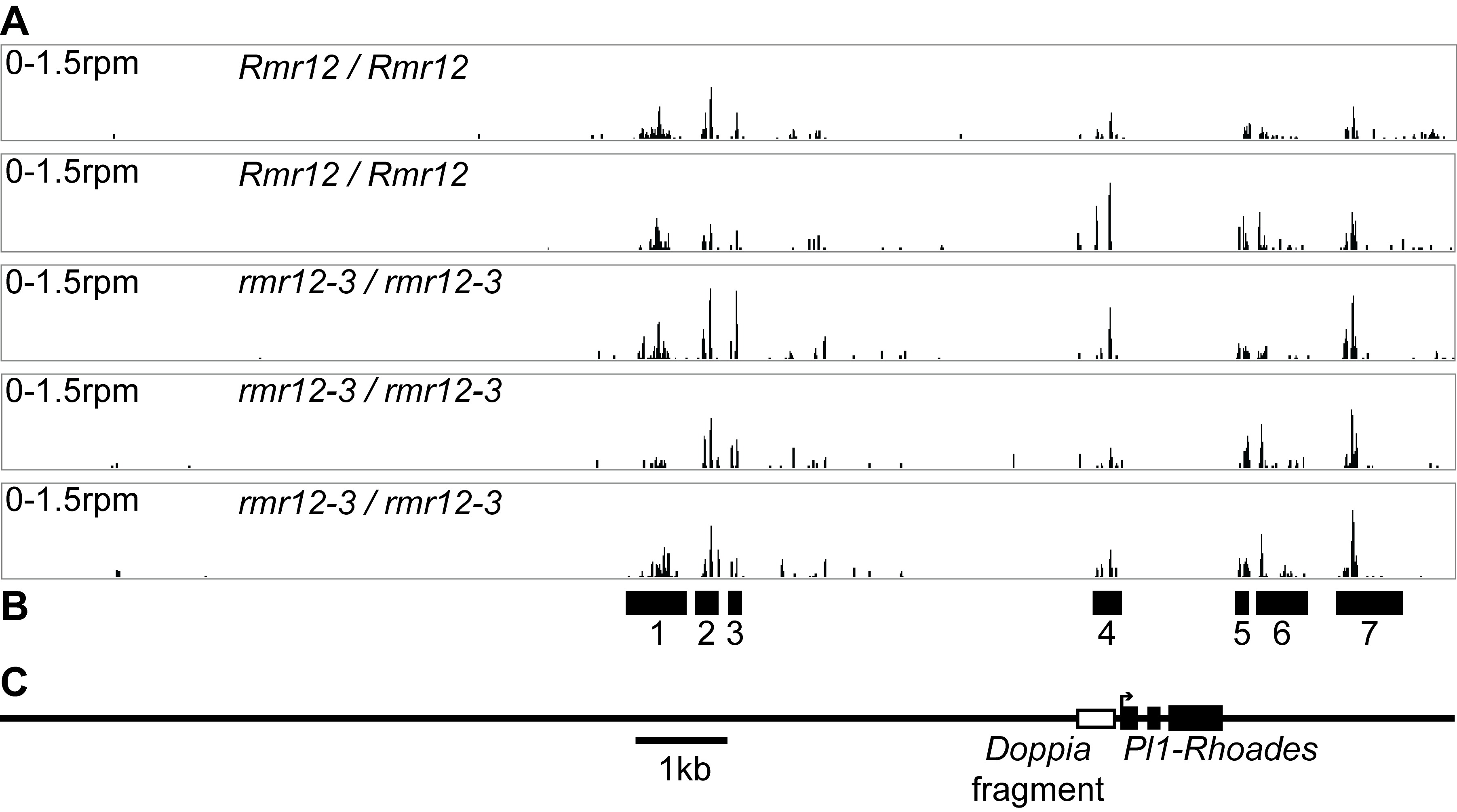

Supplement: S8 Fig — (A) Uniquely-mapping sRNA reads from each library aligned to a lambda clone sequence containing the Pl1-Rhoades coding region. Peak heights are scaled to library depth. (B) Clusters called by ShortStack with the relative position of the Pl1-Rhoades coding region (C). (TIF) [file pgen.1009243.s008.tif]
