# Supplementary material for: Locus-specific paramutation in Zea mays is maintained by a PICKLE-like chromodomain helicase DNA-binding 3 protein controlling development and male gametophyte function
Source: PLoS Genet. 2020 Dec 15;16(12):e1009243. doi: 10.1371/journal.pgen.1009243 (PMC7837471; doi:10.1371/journal.pgen.1009243)
Supplement: S1 Table — (DOCX) [file pgen.1009243.s009.docx]

| S1 Table. Genetic complementation tests based on anther pigments | | | | | |
| --- | --- | --- | --- | --- | --- |
| **Parental genotypes** | |  | **No. of individuals with the indicated anther color scores** | | |
| **Female** | **Male** | **Progeny ID** | **1-4 (Pl´ )** | **5-6 (intermediate)** | **7 (Pl-Rh)** |
| *+* / ems98738 | *mop1-1* / *mop1-1* | 011252 | 19 | 0 | 0 |
| *mop1-1* / *+* | *+* / ems98738 | 011616 | 16 | 0 | 0 |
| *+* / *rmr1-1* | *+* / ems98738 | 011556 | 19 | 0 | 0 |
| *+* / ems063095 | *rmr2-1* / *rmr2-1* | 170374 | 17 | 0 | 0 |
| *rmr1-3* / *rmr1-3* | *+* / ems98738 | 011808 | 15 | 0 | 0 |
| *+* / ems98738 | *rmr6-1* / *rmr6-1* | 011253 | 14 | 0 | 0 |
| *+* / *rmr6-1* | *+* / ems98738 | 011782 | 9 | 0 | 0 |
| *+* / ems98738 | *rmr7-3* / *rmr7-3* | 070165 | 30 | 0 | 0 |
| *+* / ems98738 | *+* / ems98924 | 013116 | 11 | 1 | 2 |
| *+* / ems98924 | *+* / ems98738 | 013117 | 12 | 1 | 4 |
