# Supplementary material for: Locus-specific paramutation in Zea mays is maintained by a PICKLE-like chromodomain helicase DNA-binding 3 protein controlling development and male gametophyte function
Source: PLoS Genet. 2020 Dec 15;16(12):e1009243. doi: 10.1371/journal.pgen.1009243 (PMC7837471; doi:10.1371/journal.pgen.1009243)
Supplement: S2 Table — (DOCX) [file pgen.1009243.s010.docx]

| **S2 Table. Genetic complementation tests based on developmental defects** | | | | |
| --- | --- | --- | --- | --- |
| **Parental genotypes** | |  | **No. of individuals with the indicated plant phenotype** | |
| **Female** | **Male** | **Progeny ID** | **Non-mutant** | **Mutant** |
| *+* / ems063095 | ems98738 / ems98738 | 142785 | 90 | 84 |
| *+* / ems063095 | ems98738 / ems98738 | 142783 | 68 | 66 |
| ems98738 / ems98738 | *+* / ems063095 | 142763 | 86 | 60 |
| ems98738 / ems98738 | *+* / ems063095 | 142850 | 42 | 21 |
| + / ems063095 | + / ems143190 | 160210 | 42 | 7 |
