# Supplementary material for: Locus-specific paramutation in Zea mays is maintained by a PICKLE-like chromodomain helicase DNA-binding 3 protein controlling development and male gametophyte function
Source: PLoS Genet. 2020 Dec 15;16(12):e1009243. doi: 10.1371/journal.pgen.1009243 (PMC7837471; doi:10.1371/journal.pgen.1009243)
Supplement: S3 Table — (DOCX) [file pgen.1009243.s011.docx]

| **S3 Table. F_2_ *rmr12* mutant frequencies** | | | | | | | | |
| --- | --- | --- | --- | --- | --- | --- | --- | --- |
| **Progeny** | | **No. individuals** | |  | **Statistics for single**  **locus model (0.25)** | | **Statistics for two**  **locus model (0.0625)** | |
| **ID** | **Allele** | **Mutant** | **Non-mutant** | **Freq.** | **Mutant χ^2^** | ***p* value** | **Mutant χ^2^** | ***p* value** |
| 013110 | *rmr12-1* | 10 | 55 | 0.15 | 2.40 | 0.08 | 8.68 | 0.002 |
| 013120 | *rmr12-1* | 20 | 84 | 0.19 | 1.38 | 0.17 | 28.04 | 6.1e-8 |
| 023745 | *rmr12-1* | 1 | 26 | 0.04 | 4.90 | 0.02 | 0.28 | 0.66 |
| 023747 | *rmr12-1* | 2 | 17 | 0.11 | 1.59 | 0.143 | 0.56 | 0.41 |
| 062104 | *rmr12-1* | 6 | 36 | 0.14 | 1.93 | 0.11 | 4.34 | 0.02 |
| 062106 | *rmr12-1* | 4 | 14 | 0.22 | 0.06 | 1.65 | 7.35 | 0.004 |
| 062111 | *rmr12-1* | 5 | 7 | 0.42 | 1.33 | 0.18 | 24.08 | 4.8e-7 |
| 062113 | *rmr12-1* | 10 | 22 | 0.31 | 0.50 | 0.44 | 32.00 | 7.9e-9 |
| 062556 | *rmr12-1* | 10 | 60 | 0.14 | 3.21 | 0.05 | 7.23 | 0.004 |
| 062557 | *rmr12-1* | 3 | 53 | 0.05 | 8.64 | 0.002 | 0.07 | 1.44 |
| 062562 | *rmr12-1* | 4 | 61 | 0.06 | 9.23 | 0.001 | 0.00 | 12.86 |
| 120527 | *rmr12-1* | 6 | 30 | 0.17 | 1.00 | 0.24 | 6.25 | 0.01 |
| 120528 | *rmr12-1* | 4 | 17 | 0.19 | 0.30 | 0.63 | 5.50 | 0.01 |
| 120530 | *rmr12-1* | 9 | 40 | 0.18 | 0.86 | 0.28 | 11.51 | 3.7e-7 |
| 120531 | *rmr12-1* | 4 | 38 | 0.10 | 4.02 | 0.03 | 0.72 | 0.33 |
| 120535 | *rmr12-1* | 3 | 17 | 0.15 | 0.80 | 0.30 | 2.45 | 0.08 |
| 120536 | *rmr12-1* | 3 | 38 | 0.07 | 5.13 | 0.01 | 0.07 | 1.40 |
| 120537 | *rmr12-1* | 7 | 27 | 0.21 | 0.26 | 0.68 | 11.18 | 4.5e-4 |
| 013114 | *rmr12-2* | 4 | 48 | 0.08 | 6.23 | 0.007 | 0.17 | 0.88 |
| 013115 | *rmr12-2* | 21 | 123 | 0.15 | 6.25 | 0.007 | 16.00 | 3.3e-5 |
| 091061 | *rmr12-3* | 7 | 17 | 0.29 | 0.17 | 0.90 | 20.17 | 1.4e-4 |
| 091069 | *rmr12-3* | 5 | 19 | 0.21 | 0.17 | 0.90 | 8.17 | 0.002 |
| 091072 | *rmr12-3* | 5 | 19 | 0.21 | 0.17 | 0.90 | 8.17 | 0.002 |
| 091073 | *rmr12-3* | 3 | 25 | 0.11 | 2.29 | 0.08 | 0.89 | 0.27 |
| 091079 | *rmr12-3* | 3 | 12 | 0.20 | 0.15 | 0.96 | 4.54 | 0.02 |
| 091103 | *rmr12-3* | 4 | 22 | 0.15 | 0.96 | 0.25 | 3.47 | 0.04 |
| 091104 | *rmr12-3* | 3 | 22 | 0.12 | 1.69 | 0.13 | 1.32 | 0.18 |
| 091126 | *rmr12-3* | 6 | 23 | 0.21 | 0.22 | 0.77 | 9.67 | 0.001 |
| 091132 | *rmr12-3* | 6 | 22 | 0.21 | 0.14 | 0.98 | 10.32 | 7.1e-4 |
| 091133 | *rmr12-3* | 23 | 103 | 0.18 | 2.29 | 0.08 | 29.05 | 3.6e-8 |
| 140036 | *rmr12-3* | 52 | 128 | 0.29 | 1.09 | 0.22 | 147.61 | 2.9e-34 |
| 140037 | *rmr12-3* | 35 | 259 | 0.12 | 20.17 | 3.7e-6 | 15.04 | 5.6e-5 |
| 141179^a^ | *rmr12-3* | 35 | 157 | 0.18 | 3.52 | 0.04 | 44.08 | 1.6e-11 |
| 141181^a^ | *rmr12-3* | 45 | 151 | 0.23 | 0.33 | 0.59 | 87.56 | 4.1e-21 |
| 141182^a^ | *rmr12-3* | 41 | 155 | 0.21 | 1.31 | 0.18 | 67.47 | 1.1e-16 |
| 141183^a^ | *rmr12-3* | 30 | 158 | 0.16 | 6.15 | 0.007 | 28.35 | 5.2e-8 |
| Totals | *rmr12-1* | 111 | 642 | 0.15 | 31.70 | 9.3e-9 | 86.86 | 5.9e-21 |
|  | *rmr12-2* | 25 | 171 | 0.13 | 11.76 | 3.3e-4 | 13.27 | 1.4e-4 |
|  | *rmr12-3* | 303 | 1292 | 0.19 | 22.99 | 8.5e7 | 414.66 | 1.8e-92 |
|  | All | 439 | 2105 | 0.18 | 61.02 | 2.9e-15 | 493.08 | 1.5e-109 |
| ^a^Phenotype scored in seedlings | | | | | | | | |
