# Supplementary material for: Locus-specific paramutation in Zea mays is maintained by a PICKLE-like chromodomain helicase DNA-binding 3 protein controlling development and male gametophyte function
Source: PLoS Genet. 2020 Dec 15;16(12):e1009243. doi: 10.1371/journal.pgen.1009243 (PMC7837471; doi:10.1371/journal.pgen.1009243)
Supplement: S4 Table — (DOCX) [file pgen.1009243.s012.docx]

| **S4 Table. *rmr12-1* and *wx1* cosegregation in progenies from self-pollinated *Rmr12* / *rmr12-1* ; *Wx1* / *wx1* plants** | | |
| --- | --- | --- |
| **Progeny** | **No. individuals of given type from *wx1* kernels** | |
| **ID** | **Non-mutant** | **Mutant** |
| 033469 | 4 | 7 |
| 033472 | 3 | 3 |
| 033473 | 10 | 3 |
| 033474 | 5 | 11 |
| 033475 | 5 | 11 |
| 033476 | 6 | 10 |
| 033478 | 8 | 11 |
| 033479 | 12 | 8 |
| 033480 | 12 | 9 |
| 033482 | 14 | 11 |
| 033483 | 8 | 9 |
| Total | 87 | 93 |
| χ^2^= 21.10, *p*<0.001. | | |
