# Supplementary material for: Locus-specific paramutation in Zea mays is maintained by a PICKLE-like chromodomain helicase DNA-binding 3 protein controlling development and male gametophyte function
Source: PLoS Genet. 2020 Dec 15;16(12):e1009243. doi: 10.1371/journal.pgen.1009243 (PMC7837471; doi:10.1371/journal.pgen.1009243)
Supplement: S8 Table — (DOCX) [file pgen.1009243.s016.docx]

| **S8 Table. *Pl1-Rhoades* proximal sRNA cluster representation in *rmr12* mutants** | | | | | | | |
| --- | --- | --- | --- | --- | --- | --- | --- |
| **Cluster** | **Position (bp)** | ***Rmr12*** | ***Rmr12*** | ***rmr12-3*** | ***rmr12-3*** | ***rmr12-3*** | ***p-value**** |
| 1 | 6919-7587 | 2.00 | 1.80 | 2.30 | 0.89 | 2.09 | 0.78 |
| 2 | 7687-7939 | 1.50 | 0.81 | 1.89 | 1.59 | 1.72 | 0.33 |
| 3 | 8047-8203 | 0.52 | 0.45 | 1.37 | 0.84 | 0.59 | 0.18 |
| 4 | 12078-12402 | 0.77 | 1.76 | 1.15 | 0.70 | 0.97 | 0.63 |
| 5 | 13644-13796 | 0.67 | 1.26 | 0.85 | 1.12 | 1.03 | 0.92 |
| 6 | 13880-14455 | 0.67 | 1.49 | 0.85 | 1.64 | 1.38 | 0.70 |
| 7 | 14761-15499 | 1.31 | 1.89 | 2.45 | 2.20 | 2.25 | 0.23 |
| Counts represent uniquely-mapping 18-30nt reads per million clean reads. *2-sample *t*-test | | | | | | | |
