# Supplementary material for: Locus-specific paramutation in Zea mays is maintained by a PICKLE-like chromodomain helicase DNA-binding 3 protein controlling development and male gametophyte function
Source: PLoS Genet. 2020 Dec 15;16(12):e1009243. doi: 10.1371/journal.pgen.1009243 (PMC7837471; doi:10.1371/journal.pgen.1009243)
Supplement: S9 Table — (DOCX) [file pgen.1009243.s017.docx]

| **S9 Table. Primers used in this study** | | |
| --- | --- | --- |
| **ID** | **Sequence** | **Restriction enzyme** |
| **Molecular markers** | | |
| 9_12.38 F | GGTTTGACGAATCAAGGCGG | *Sau*96I |
| 9_12.38 R | GCAGCCCATCGAGCAATTCT |  |
| 9_16.47 F | GATTCTTCTGTGCTGGAAAGTCATCA | *Hin*dIII |
| 9_16.47 R | CCGAACCACTAAAATGCTTCGC**[A]**A |  |
| 9_50.02 F | TGCTCATCGAATCGGGAAGA | *Hin*cII |
| 9_50.02 R | GAAGCAACACGAACCTGCAG |  |
| 9_61.09 F | CGAAAGAGGCCGATCCATCTGA**[A]**TT | *Apo*I |
| 9_61.09 R | TGAGAGCGTGGAAGTTGCAT |  |
| 9_87.31 F | CCACGGACTGGTGCAGTATC | *Tfi*I |
| 9_87.31 R | ATGGCCGTATGGACCAAAGG |  |
| ***rmr12-3* intron inclusion** | | |
| FP | ATTGATGGAGGGCGAAGG |  |
| RP | CTCTGGATTTCCTCGACACTTT |  |
| **Genotyping** | | |
| *rmr12-3* F | CAATGTTCCTACAGAC**[C]**CTCAATAG | *Bsl*I |
| *rmr12-3* R | CTGTGTGCCTGCTTTATCACG |  |
| *rmr12-4* F | TGAACGCATCGATGGCAAGA | *Mwo*I |
| *rmr12-4* R | GTCATCCCAGACAGAAGGTACT |  |
| **qRT-PCR** |  |  |
| *pl1_33_F* | GAAAGAGAGAGAGAGCGAGAGATGGGC |  |
| *pl1_143_R* | CTTGACGTAGGCGGCCAAGGTATC |  |
| *gapdh_F* | CCTGCTTCTCATGGATGGTT |  |
| *gapdh_R* | TGGTAGCAGGAAGGGAAACA |  |
| **Sanger sequencing** | | |
| *chd3*-seq F1 | CGCTAGAGGTGGAACGAGAC |  |
| *chd3*-seq R1 | GCCCTTGTCACCAGATTTCTTAC |  |
| *chd3*-seq F2 | CTGGAAGCACCTCGTGAAGA |  |
| *chd3*-seq R2 | AGTTGCAAATTCACGCTCCC |  |
| *chd3*-seq F3 | ACAAACGCGTAATCCTTGGTG |  |
| *chd3*-seq R3 | AGCTCTTGCCATAGCTTGCA |  |
| *chd3*-seq F4 | AGCTGGTGGTCTGGGAATAAA |  |
| *chd3*-seq R4 | GGATTTCCTCGACACTTTTTCCT |  |
| *chd3*-seq F5 | GGATTCAACCATGCTCAACG |  |
| *chd3*-seq R5 | TGCACCTTCCAAATTCCCAT |  |
| *chd3*-seq F6 | GGTGCTCAGTTGAACGAGGC |  |
| *chd3*-seq R6 | TGTAACTGACCAGCTGTGCC |  |
| *chd3*-seq F7 | GGCCAAGCGAACACAACAAG |  |
| *chd3*-seq R7 | TAGCGCACATTCTGACCACA |  |
| **[ ]**: adapted to introduce enzyme recognition sequence. | | |
