# Supplementary material for: Locus-specific paramutation in Zea mays is maintained by a PICKLE-like chromodomain helicase DNA-binding 3 protein controlling development and male gametophyte function
Source: PLoS Genet. 2020 Dec 15;16(12):e1009243. doi: 10.1371/journal.pgen.1009243 (PMC7837471; doi:10.1371/journal.pgen.1009243)
Supplement: S10 Table — (DOCX) [file pgen.1009243.s018.docx]

| **S10 Table. sRNA library statistics** | | | | | | | | |
| --- | --- | --- | --- | --- | --- | --- | --- | --- |
| **Genotype** | **Raw reads** | **Clean reads*** | **Uniquely mapped** | | | | **Unmapped** | **Multiply-mapped** |
|  |  |  | **All** | **21** | **22** | **24** |  |  |
| *Rmr12/Rmr12* | 231 M | 79 M | 12.4 M | 0.9 M | 1.1 M | 7.7 M | 12.8 M | 54.2 M |
| *Rmr12/Rmr12* | 69 M | 22 M | 4.0 M | 0.3 M | 0.3 M | 2.6 M | 4.0 M | 14.2 M |
| *rmr12-3/rmr12-3* | 65 M | 27 M | 6.0 M | 0.4 M | 0.5 M | 4.0 M | 5.6 M | 15.4 M |
| *rmr12-3/rmr12-3* | 56 M | 21 M | 4.2 M | 0.3 M | 0.3 M | 2.7 M | 4.1 M | 13.1 M |
| *rmr12-3/rmr12-3* | 95 M | 32 M | 6.5 M | 0.4 M | 0.5 M | 4.3 M | 6.2 M | 19.3 M |
| Total | 516 M | 182 M | 33.1 M | 2.4 M | 2.7 M | 21.3 M | 32.6 M | 116.3 M |
| *18-30nt; M: million reads | | | | | | | | |
