# Supplementary material for: Locus-specific paramutation in Zea mays is maintained by a PICKLE-like chromodomain helicase DNA-binding 3 protein controlling development and male gametophyte function
Source: PLoS Genet. 2020 Dec 15;16(12):e1009243. doi: 10.1371/journal.pgen.1009243 (PMC7837471; doi:10.1371/journal.pgen.1009243)
Supplement: S1 Methods — (DOCX) [file pgen.1009243.s019.docx]

**Supplemental methods**

**Genetic materials**

Inbred lines A619, A632, and B73 were sourced from the North Central Regional Plant Introduction Station (USDA-ARS, Ames, IA). The *Pl1-Rhoades* haplotype introgressed into A619 and A632 [1] came from a previously described color-converted W23 stock (herein designated W23 *Pl1-Rh*) [2] developed by Ed Coe, Jr. (USDA-ARS, University of Missouri, Columbia, MO) and maintained in several lines obtained from the Maize Genetics Cooperation Stock Center (MGCSC) (USDA-ARS, University of Illinois, Urbana, IL) and Vicki Chandler (University of Arizona, Tucson, AZ) including a previously described *Pl1-Rhoades*-converted K55 line [3]. The A632 *Pl1-Rh* line used in this study has a recessive *r1* derivative of the original *R1-A632* allele that promotes anthocyanin production in the anthers but not in the kernel. A *T6-9 (043-1)* [4] interchange chromosome (*T Pl1-Rh*) having psuedolinkage between *Pl1-Rhoades* and a recessive *waxy1* allele (*wx1*) was introgressed into B73 (herein designated B73 *T Pl1-Rh*) as previously described [1,5], and into A619 (herein designated A619 *T Pl1-Rh* ; ~97% A619), A632 (herein designated A632 *T Pl1-Rh* ; ~98% A632), and Mo17 (herein designated Mo17 *T Pl1-Rh* ; ~97% Mo17). The *c1* tester used in this study was generated by crossing a recessive MGCSC stock 905H (*c1 sh1 wx1*) to W23 *Pl1-Rh* (*C1 / C1*) and selecting colorless F_2_ kernels.

**Evaluated progenies.** Images of *rmr12-3* mutant and non-mutant sibling adult plants and seedlings were from F_2_ progenies 141174 and 141180, respectively. The imaged *rmr12-3* mutant tassel was from F_2_ progeny 141178, and the imaged ears representing reciprocal crosses between A619 *Pl1-Rh* and a single *rmr12-1* mutant hold progenies 141250 and 142522. The imaged leaf from Fig 1C was from progeny 141171, and two leaves from S1A Fig were from 141180 while the third was from 141176. The leaves from S1B Fig were from progenies 180001, 180002, and 143190. The imaged *B-I* and *B´* individuals were from progenies 180001 and 180002 respectively. The imaged F_2_ ear holds progeny 170153, and the imaged test cross ear holds progeny 170323. The *rmr12* mutant frequencies were recorded in F_2_ progenies segregating *rmr12-1* (013110, 013120, 023745, 023747, 062104, 062106, 062111, 062113, 062556, 062557, 062562, 120527, 120528, 120530, 120531, 120535 - 120537), *rmr12-2* (013114, 013115), and *rmr12-3* (091061, 091069, 091072, 091073, 091079, 091103, 091104, 091126, 091132, 091133, 140036, 140037, 141179, 141181 - 141183). The effect of *rmr12* mutations on recessive *pl1* alleles was evaluated in one F_2_ progeny segregating *rmr12-1* and *pl1-A632* (013120) and one progeny segregating *rmr12-2* and *pl1-B73* (013115). Paramutagenicity of sexually transmitted *Pl1-Rhoades* alleles was measured in 11 testcrossed progenies from homozygous *rmr12-3* mutants (172608, 172609, 172611, 172612, 172613, 172614, 172615, 172619, 172620), one testcrossed progeny from a *rmr12-3 / rmr12-4* heterozygote (172618), and two testcrossed progenies from homozygous *rmr12-4* individuals (160777, and 160778). Genetic linkage between *rmr12-1* and *wx1* was measured in 11 progenies (033469, 033472-033476, 033478-033480, 033482, 033483). The *rmr12-3* mutants used for recombinant-based mapping came from progenies 141167, 141171 - 141174, 141176, 141178 - 141183. sRNAs for bulk sRNA profiles in S7 Fig were isolated from seedlings in progeny 170143, and sRNA libraries were prepared from individuals in progeny 181977. qRT-PCR was done on individuals from progeny 181978. Plant height and days to flowering were measured in three F_2_ progenies (013110, 013115, 091133) segregating *rmr12-1*, *rmr12-2*, and *rmr12-3* respectively. Internode length was measured in one *rmr12-3* F_2_ progeny (150005). Juvenile to adult transition was recorded in progeny 170380. Leaf length and width were measured in two *rmr12-3* F_2_ progenies (140036 and 140037). Secondary veins were counted on individuals from three *rmr12-3* F_2_ progenies (141171, 141174, 141176). Primary spike length and numbers of tassel branches and ear shoots were measured in eight *rmr12-3* F_2_ progenies (141167, 141171, 141174, 141178, 141180, 170027, 170028, 170029). Mutant frequencies were recorded from four progenies (142763, 142783, 142785, 142850) derived from crosses between *rmr12-1* mutants and *rmr12-3* heterozygotes. Biased transmission of the dominant *C1* allele linked to *rmr12-4* was observed in 9 testcross progenies (170107, 170118, 170122, 170124, 170131, 170134, 170323, 170324, 170782). Frequency of glassy and waxy pollen types was determined in heterozygotes for *rmr12-3* and *wx1* from progeny 160372. *In vitro* pollen germination and viability staining was measured in individuals from progeny 180526.

***rmr12-1* pedigrees.** The *rmr12-1* allele was identified in a mutant screen using a mixed genetic background as previously described (Hollick and Chandler, 2001). Mutants from the *rmr12-1* M_2_ progeny (designated 98738) were crossed to both A632 and B73 and respective F_1_ individuals were self-pollinated to generate F_2_ progenies 013110 and 013120. A single mutant from 013110 was crossed to *T* *Pl1-Rh*, and resulting F_1_ plants were self-pollinated to generate progenies 023745 and 023747. A single F_2_ mutant from 023747 was backcrossed to A632 and resulting heterozygotes were self-pollinated to generate progenies 033469, 033472 - 033476, 033478 - 033480, and 033482 - 033483. A subsequent backcross and self-pollination generated a BC_2_F_2_ individual that was crossed to A632 *Pl1-Rh*. BC_3_F_2_ progenies include 062104, 062106, 062111, 062113, 062556, 062557, and 062562. BC_3_F_2_ heterozygotes were self-pollinated to generate progenies 120527, 120528, 120530, 120531, 120535, 120536, and 120537. A mutant from 120527 was crossed to A619 *T* *Pl1-Rh* and a resulting F_1_ was self-pollinated. F_2_ mutants were backcrossed to and by A619 *T* *Pl1-Rh* to generate progenies 141250 and 142522 respectively.

***rmr12-2* pedigrees.** The *rmr12-2* allele was identified in the same mutant screen as *rmr12-1*. A single mutant from the M_2_ progeny (098924) was crossed to A632 and two F_1_ heterozygotes were self-pollinated to generate progenies 013114 and 013115. To test complementation between *rmr12* alleles, an *Rmr12-B73* / *rmr12-1* F_1_ individual was crossed by and to an *Rmr12-A632* / *rmr12-2* F_1_ individual generating progenies 013116 and 013117 respectively.

***rmr12-3* pedigrees.** The *rmr12-3* allele was identified in a previously described mutant screen (Hale et al., 2007) in which ethyl methanesulfonate-treated pollen from an A619 *Pl1-Rh* (*Pl-Rh*) line was crossed to A632 *Pl1-Rh* (*Pl´*). Heterozygotes from the *rmr12-3* M_2_ progeny (063095) were backcrossed to A632 *Pl1-Rh* (*Pl´*) and BC_1_ heterozygotes were self-pollinated to generate F_2_ progenies 091061, 091069, 091072, 091073, 091079, 091103, 091104, 091126, 091132, and 091133. A single BC_1_F_2_ mutant was backcrossed by A632 *Pl1-Rh*, and two resulting heterozygotes were both self-pollinated to generate progenies 140036 and 140037 and backcrossed to A632 *Pl1-Rh*. Eleven BC_3_F_1_ plants were self-pollinated to generate F_2_ progenies 141167, 141171, 141172, 141173, 141174, 141176, 141178, 141179, 141180, 141181, 141182, and 141183. The introgression into A632 *Pl1-Rh* (*Pl´*) continued using the same strategy to generate BC_4_F_2_ progeny 150005, BC_5_F_2_ progenies 170027-170029, and 170380, and BC_6_F_2_ progenies 181977 and 181978. An A632 *Pl1-Rh* BC_2_ *rmr12-3* heterozygote was crossed to B73 *T* *Pl1-Rh*, and, by repeatedly backcrossing heterozygotes, the allele was introgressed into B73 *T* *Pl1-Rh* to generate the BC_3_F_2_ progeny 160372. To test genetic complementation, homozygous *rmr12-1* mutants from two A619 *Pl1-Rh* F_2_ progenies were crossed to and by A632 *Pl1-Rh* BC_1_F_2_ *rmr12-3* heterozygotes to generate four progenies (142783, 142785, 142763, and 142850). Three A632 *Pl1-Rh* BC_3_F_2_ *rmr12-3* mutants were testcrossed by B73 *T* *Pl1-Rh (Pl-Rh)* to generate progenies 172608, 172613, and 172620. Four A632 *Pl1-Rh* BC_3_F_2_ *rmr12-3* mutants were testcrossed by W23 *Pl1-Rh* (*Pl-Rh*) to generate progenies 172609, 172612, 172614, and 172619. Two A632 *Pl1-Rh* BC_3_F_2_ *rmr12-3* mutants were testcrossed by A632 *T* *Pl1-Rh (Pl-Rh)* and W23 *Pl1-Rh* (*Pl-Rh*) to generate progenies 172611 and 172615 respectively.

***rmr12-4* pedigrees.** The *rmr12-4* allele was identified in the same screen as *rmr12-3*. A heterozygote from the *rmr12-*4 M_2_ progeny (143190) was crossed to an *rmr12-3* heterozygote from the 160210 progeny that resulted from crossing an A632 *Pl1-Rh* *rmr12-3* BC_2_F_2_ mutant by a BC_2_ heterozygote. An *rmr12-3 / rmr12-4* mutant was crossed by B73 *T* *Pl1-Rh (Pl-Rh)* to generate progeny 172618. Two M_2_ mutants were crossed by W23 *Pl1-Rh* (*Pl-Rh*) to generate progenies 160777 and 160778. M_2_ heterozygotes were self-pollinated to generate M_2_F_2_ individuals; two of these were self-pollinated to generate progenies 170143 and 170153. Four M_2_F_2_ heterozygotes were crossed to A632 *Pl1-Rh* (*Pl´*) generating progenies 170107, 170118, 170122, and 170124, and two heterozygotes were crossed to a recessive *c1* stock to generate progenies 170323 and 170324. Two M_2_F_2_ heterozygotes were crossed to Mo17 *T Pl1-Rh* to generate progenies 170131 and 170134. An A632 *Pl1-Rh* *rmr12-4* BC_2_F_2_ heterozygote was crossed to A632 *Pl1-Rh* (*Pl´*) to generate progeny 170782. A M_2_F_2_ heterozygote was crossed to a recessive *c1* stock that also carried recessive *wx1* to generate 180526. The progenies used to evaluate *b1* paramutation in *rmr12-4* mutants (180787-180789) were generated by crossing *rmr12-4 / rmr12-4* individuals from progenies 180001, 180003, and 180005 to recessive A619 K55 *b1* testers. To ensure the mutants evaluated were not carrying a recessive *b1* allele, only testcross progenies which contained at least five individuals and no colorless plants were included in Table 10. To generate progenies 180001 and 180005, *B1-I* ( *B´* ) / *b1*; *Rmr12* / *rmr12-4* individuals from progeny 170887 were crossed by *B1-I* ( *B-I* ) / *B1-I* ( *B-I* ); *Rmr12* / *rmr12-4* individuals from progeny 170877. To generate progeny 180002 and 180003, *B1-I* ( *B´* ) / *B1-I* ( *B´* ); *Rmr12* / *rmr12-4* individuals from progeny 170887 were crossed by *B1-I* ( *B-I* ) / *b1*; *Rmr12* / *rmr12-4* individuals from progeny 170877. Progeny 170887 was generated by crossing a *B1-I* ( *B´* ) / *b1*; *Rmr12* / *rmr12-4* M_2_ individual to W23 *B1-I* ( *B´* ), and progeny 170877 resulted from crossing a *B1-I* ( *B-I* ) / *b1*; *Rmr12* / *rmr12-4* A632 *Pl1-Rh* BC_1_F_2_ individual to W23 *B1-I* ( *B-I* ).

**Genetic linkage**

Kernels having opaque endosperms (diagnostic of *wx1* mutants) selected from 11 progenies generated by self-pollinating *Rmr12* / *rmr12-1* ; *Wx1* / *wx1* individuals gave rise to 52% *rmr12-1* mutants (see S4 Table) which deviated significantly (*χ^2^*= 21.10, *p*<0.001) from the expected frequencies if the two loci were unlinked. The overabundance of *rmr12-1* mutants in these selected progenies contrasted with the 0.18 frequencies found in other F_2_ families indicating that the two loci are genetically linked.

**References**

1. Hollick JB, Kermicle JL, Parkinson SE. *Rmr6* maintains meiotic inheritance of paramutant states in *Zea mays*. Genetics. 2005;171: 725–740.
2. Hollick JB, Patterson GI, Coe EH, Cone KC, Chandler VL. Allelic interactions heritably alter the activity of a metastable maize *pl* allele. Genetics. 1995;141: 709–719.
3. Dorweiler JE, Carey CC, Kubo KM, Hollick JB, Kermicle JL, Chandler VL. *mediator of paramutation1* is required for establishment and maintenance of paramutation at multiple maize loci. Plant Cell. 2000;12: 2101–2118. doi:10.1105/tpc.12.11.2101
4. Longley AE. Breakage points for four corn translocation series and other corn chromosome aberrations. USDA-ARS Crop Res Bull. 1961;34–16: 1–40.
5. Gross SM, Hollick JB. Multiple *trans*-sensing interactions affect meiotically heritable epigenetic states at the maize *pl1* locus. Genetics. 2007;176: 829–839. doi:10.1534/genetics.107.072496
