# Supplementary material for: Locus-specific paramutation in Zea mays is maintained by a PICKLE-like chromodomain helicase DNA-binding 3 protein controlling development and male gametophyte function
Source: PLoS Genet. 2020 Dec 15;16(12):e1009243. doi: 10.1371/journal.pgen.1009243 (PMC7837471; doi:10.1371/journal.pgen.1009243)
Supplement: S2 File — (DOCX) [file pgen.1009243.s021.docx]

**Statistic tables:**

**Figure 3A (Days to flowering)**

*rmr12-1*

t-Test: Two-Sample Assuming Unequal Variances

|  | Non-mutant | *rmr12-1* |
| --- | --- | --- |
| Mean | 82.150943 | 100.5 |
| Variance | 31.8613933 | 14.3333333 |
| Observations | 53 | 4 |
| Hypothesized Mean Difference | 0 |  |
| df | 4 |  |
| t Stat | -8.9699988 |  |
| P(T<=t) one-tail | 0.000427 |  |
| t Critical one-tail | 2.131847 |  |
| P(T<=t) two-tail | 0.00085473 |  |
| t Critical two-tail | 2.77644511 |  |

*rmr12-2*

t-Test: Two-Sample Assuming Unequal Variances

|  | Non-mutant | *rmr12-2* |
| --- | --- | --- |
| Mean | 74.48 | 102.5 |
| Variance | 13.152653 | 18.7 |
| Observations | 50 | 6 |
| Hypothesized Mean Difference | 0 |  |
| df | 6 |  |
| t Stat | -15.241503 |  |
| P(T<=t) one-tail | 2.5178E-06 |  |
| t Critical one-tail | 1.9431803 |  |
| P(T<=t) two-tail | 5.0357E-06 |  |
| t Critical two-tail | 2.4469119 |  |

*rmr12-3*

t-Test: Two-Sample Assuming Unequal Variances

|  | Non-mutant | *rmr12-3* |
| --- | --- | --- |
| Mean | 66.244898 | 89.9333333 |
| Variance | 34.0837366 | 34.352381 |
| Observations | 98 | 15 |
| Hypothesized Mean Difference | 0 |  |
| df | 19 |  |
| t Stat | -14.584883 |  |
| P(T<=t) one-tail | 4.504E-12 |  |
| t Critical one-tail | 1.72913281 |  |
| P(T<=t) two-tail | 9.008E-12 |  |
| t Critical two-tail | 2.09302405 |  |

**Figure 3B (Plant height)**

*rmr12-1*

t-Test: Two-Sample Assuming Unequal Variances

|  | Non mutant | rmr12-1 |
| --- | --- | --- |
| Mean | 212.042963 | 116.5225 |
| Variance | 369.990919 | 704.952507 |
| Observations | 54 | 8 |
| Hypothesized Mean Difference | 0 |  |
| df | 8 |  |
| t Stat | 9.80170193 |  |
| P(T<=t) one-tail | 4.9277E-06 |  |
| t Critical one-tail | 1.85954804 |  |
| P(T<=t) two-tail | 9.8553E-06 |  |
| t Critical two-tail | 2.30600414 |  |

*rmr12-2*

t-Test: Two-Sample Assuming Unequal Variances

|  | Non-mutant | *rmr12-2* |
| --- | --- | --- |
| Mean | 226.4 | 163.333333 |
| Variance | 260.653061 | 389 |
| Observations | 50 | 9 |
| Hypothesized Mean Difference | 0 |  |
| df | 10 |  |
| t Stat | 9.06189352 |  |
| P(T<=t) one-tail | 1.9453E-06 |  |
| t Critical one-tail | 1.81246112 |  |
| P(T<=t) two-tail | 3.8906E-06 |  |
| t Critical two-tail | 2.22813885 |  |

*rmr12-3*

t-Test: Two-Sample Assuming Unequal Variances

|  | *Non-mutant* | *rmr12-3* |
| --- | --- | --- |
| Mean | 184.122449 | 102.466667 |
| Variance | 291.551862 | 94.4095238 |
| Observations | 98 | 15 |
| Hypothesized Mean Difference | 0 |  |
| df | 29 |  |
| t Stat | 26.8207428 |  |
| P(T<=t) one-tail | 2.5364E-22 |  |
| t Critical one-tail | 1.69912703 |  |
| P(T<=t) two-tail | 5.0728E-22 |  |
| t Critical two-tail | 2.04522964 |  |

**Figure 3C (Internode lengths)**

Internode 1

t-Test: Two-Sample Assuming Unequal Variances

|  | Non-mutant | *rmr12-3* |
| --- | --- | --- |
| Mean | 10.0833333 | 3.91666667 |
| Variance | 11.5378788 | 2.33606061 |
| Observations | 12 | 12 |
| Hypothesized Mean Difference | 0 |  |
| df | 15 |  |
| t Stat | 5.73510271 |  |
| P(T<=t) one-tail | 1.9722E-05 |  |
| t Critical one-tail | 1.75305036 |  |
| P(T<=t) two-tail | 3.9443E-05 |  |
| t Critical two-tail | 2.13144955 |  |

Internode 2

t-Test: Two-Sample Assuming Unequal Variances

|  | Non-mutant | *rmr12-3* |
| --- | --- | --- |
| Mean | 14 | 6.20833333 |
| Variance | 12.45454545 | 2.8844697 |
| Observations | 12 | 12 |
| Hypothesized Mean Difference | 0 |  |
| df | 16 |  |
| t Stat | 6.891634709 |  |
| P(T<=t) one-tail | 1.81285E-06 |  |
| t Critical one-tail | 1.745883676 |  |
| P(T<=t) two-tail | 3.62571E-06 |  |
| t Critical two-tail | 2.119905299 |  |

Internode 3

t-Test: Two-Sample Assuming Unequal Variances

|  | Non-mutant | *rmr12-3* |
| --- | --- | --- |
| Mean | 15.4583333 | 7.34166667 |
| Variance | 7.79356061 | 1.51901515 |
| Observations | 12 | 12 |
| Hypothesized Mean Difference | 0 |  |
| df | 15 |  |
| t Stat | 9.21368636 |  |
| P(T<=t) one-tail | 7.2569E-08 |  |
| t Critical one-tail | 1.75305036 |  |
| P(T<=t) two-tail | 1.4514E-07 |  |
| t Critical two-tail | 2.13144955 |  |

Internode 4

t-Test: Two-Sample Assuming Unequal Variances

|  | Non-mutant | *rmr12-3* |
| --- | --- | --- |
| Mean | 15.2916667 | 7.66666667 |
| Variance | 2.06628788 | 1.1969697 |
| Observations | 12 | 12 |
| Hypothesized Mean Difference | 0 |  |
| df | 21 |  |
| t Stat | 14.6219331 |  |
| P(T<=t) one-tail | 8.7921E-13 |  |
| t Critical one-tail | 1.7207429 |  |
| P(T<=t) two-tail | 1.7584E-12 |  |
| t Critical two-tail | 2.07961384 |  |

Internode 5

t-Test: Two-Sample Assuming Unequal Variances

|  | Non-mutant | *rmr12-3* |
| --- | --- | --- |
| Mean | 13.9166667 | 7.01666667 |
| Variance | 3.21969697 | 0.77606061 |
| Observations | 12 | 12 |
| Hypothesized Mean Difference | 0 |  |
| df | 16 |  |
| t Stat | 11.9574933 |  |
| P(T<=t) one-tail | 1.083E-09 |  |
| t Critical one-tail | 1.74588368 |  |
| P(T<=t) two-tail | 2.166E-09 |  |
| t Critical two-tail | 2.1199053 |  |

Internode 6

t-Test: Two-Sample Assuming Unequal Variances

|  | Non-mutant | *rmr12-3* |
| --- | --- | --- |
| Mean | 14.70833333 | 6.66666667 |
| Variance | 0.884469697 | 1.28787879 |
| Observations | 12 | 12 |
| Hypothesized Mean Difference | 0 |  |
| df | 21 |  |
| t Stat | 18.90044122 |  |
| P(T<=t) one-tail | 5.79212E-15 |  |
| t Critical one-tail | 1.720742903 |  |
| P(T<=t) two-tail | 1.15842E-14 |  |
| t Critical two-tail | 2.079613845 |  |

Internode 7

t-Test: Two-Sample Assuming Unequal Variances

|  | Non-mutant | *rmr12-3* |
| --- | --- | --- |
| Mean | 12.775 | 6 |
| Variance | 2.93477273 | 1.63636364 |
| Observations | 12 | 12 |
| Hypothesized Mean Difference | 0 |  |
| df | 20 |  |
| t Stat | 10.9771054 |  |
| P(T<=t) one-tail | 3.2265E-10 |  |
| t Critical one-tail | 1.72471824 |  |
| P(T<=t) two-tail | 6.4529E-10 |  |
| t Critical two-tail | 2.08596345 |  |

Internode 8

t-Test: Two-Sample Assuming Unequal Variances

|  | Non-mutant | *rmr12-3* |
| --- | --- | --- |
| Mean | 13.0833333 | 5.275 |
| Variance | 0.90151515 | 2.26204545 |
| Observations | 12 | 12 |
| Hypothesized Mean Difference | 0 |  |
| df | 19 |  |
| t Stat | 15.2076073 |  |
| P(T<=t) one-tail | 2.1604E-12 |  |
| t Critical one-tail | 1.72913281 |  |
| P(T<=t) two-tail | 4.3208E-12 |  |
| t Critical two-tail | 2.09302405 |  |

Internode 9

t-Test: Two-Sample Assuming Unequal Variances

|  | Non-mutant | *rmr12-3* |
| --- | --- | --- |
| Mean | 12.7916667 | 4.66666667 |
| Variance | 1.20265152 | 3.37878788 |
| Observations | 12 | 12 |
| Hypothesized Mean Difference | 0 |  |
| df | 18 |  |
| t Stat | 13.1496145 |  |
| P(T<=t) one-tail | 5.7077E-11 |  |
| t Critical one-tail | 1.73406361 |  |
| P(T<=t) two-tail | 1.1415E-10 |  |
| t Critical two-tail | 2.10092204 |  |

Internode 10

t-Test: Two-Sample Assuming Unequal Variances

|  | Non-mutant | *rmr12-3* |
| --- | --- | --- |
| Mean | 12.21666667 | 4.55 |
| Variance | 1.71969697 | 3.61181818 |
| Observations | 12 | 12 |
| Hypothesized Mean Difference | 0 |  |
| df | 20 |  |
| t Stat | 11.50196073 |  |
| P(T<=t) one-tail | 1.43232E-10 |  |
| t Critical one-tail | 1.724718243 |  |
| P(T<=t) two-tail | 2.86465E-10 |  |
| t Critical two-tail | 2.085963447 |  |

Internode 11

t-Test: Two-Sample Assuming Unequal Variances

|  | Non-mutant | *rmr12-3* |
| --- | --- | --- |
| Mean | 12.0555556 | 4.69166667 |
| Variance | 4.34027778 | 3.96628788 |
| Observations | 9 | 12 |
| Hypothesized Mean Difference | 0 |  |
| df | 17 |  |
| t Stat | 8.16810861 |  |
| P(T<=t) one-tail | 1.3734E-07 |  |
| t Critical one-tail | 1.73960673 |  |
| P(T<=t) two-tail | 2.7467E-07 |  |
| t Critical two-tail | 2.10981558 |  |

Internode 12

t-Test: Two-Sample Assuming Unequal Variances

|  | Non-mutant | *rmr12-3* |
| --- | --- | --- |
| Mean | 12.2 | 4.90909091 |
| Variance | 0.825 | 5.84090909 |
| Observations | 5 | 11 |
| Hypothesized Mean Difference | 0 |  |
| df | 14 |  |
| t Stat | 8.73936025 |  |
| P(T<=t) one-tail | 2.4116E-07 |  |
| t Critical one-tail | 1.76131014 |  |
| P(T<=t) two-tail | 4.8231E-07 |  |
| t Critical two-tail | 2.14478669 |  |

Internode 13

t-Test: Two-Sample Assuming Unequal Variances

|  | Non-mutant | *rmr12-3* |
| --- | --- | --- |
| Mean | 11 | 3.75 |
| Variance | #DIV/0! | 3.791 |
| Observations | 1 | 6 |
| Hypothesized Mean Difference | 0 |  |
| df | 65535 |  |
| t Stat | 9.12087995 |  |
| P(T<=t) one-tail | #NUM! |  |
| t Critical one-tail | #NUM! |  |
| P(T<=t) two-tail | #NUM! |  |
| t Critical two-tail | #NUM! |  |

**Figure 3D (Transition leaves)**

t-Test: Two-Sample Assuming Unequal Variances

|  | Non-mutant | *rmr12-3* |
| --- | --- | --- |
| Mean | 5.66666667 | 6.77777778 |
| Variance | 0.25 | 0.94444444 |
| Observations | 9 | 9 |
| Hypothesized Mean Difference | 0 |  |
| df | 12 |  |
| t Stat | -3.0499714 |  |
| P(T<=t) one-tail | 0.00504263 |  |
| t Critical one-tail | 1.78228756 |  |
| P(T<=t) two-tail | 0.01008525 |  |
| t Critical two-tail | 2.17881283 |  |

**Figure 4A (Leaf lengths)**

t-Test: Two-Sample Assuming Unequal Variances

|  | Non-mutant | *rmr12-3* |
| --- | --- | --- |
| Mean | 61.5864407 | 57.4736842 |
| Variance | 63.0878154 | 200.093883 |
| Observations | 236 | 38 |
| Hypothesized Mean Difference | 0 |  |
| df | 41 |  |
| t Stat | 1.74845569 |  |
| P(T<=t) one-tail | 0.04393496 |  |
| t Critical one-tail | 1.682878 |  |
| P(T<=t) two-tail | 0.08786991 |  |
| t Critical two-tail | 2.01954097 |  |

**Figure 4B (Leaf widths)**

t-Test: Two-Sample Assuming Unequal Variances

|  | Non-mutant | *rmr12-3* |
| --- | --- | --- |
| Mean | 5.24279661 | 3.32894737 |
| Variance | 1.24135215 | 0.88211238 |
| Observations | 236 | 38 |
| Hypothesized Mean Difference | 0 |  |
| df | 55 |  |
| t Stat | 11.3419486 |  |
| P(T<=t) one-tail | 2.5336E-16 |  |
| t Critical one-tail | 1.67303397 |  |
| P(T<=t) two-tail | 5.0672E-16 |  |
| t Critical two-tail | 2.00404478 |  |

**Figure 4C (No. lateral veins)**

t-Test: Two-Sample Assuming Unequal Variances

|  | Non-mutant | *rmr12-3* |
| --- | --- | --- |
| Mean | 20.8 | 17.1666667 |
| Variance | 0.84444444 | 3.76666667 |
| Observations | 10 | 6 |
| Hypothesized Mean Difference | 0 |  |
| df | 6 |  |
| t Stat | 4.30524116 |  |
| P(T<=t) one-tail | 0.00253184 |  |
| t Critical one-tail | 1.94318028 |  |
| P(T<=t) two-tail | 0.00506369 |  |
| t Critical two-tail | 2.44691185 |  |

**Figure 4D (No. tassel branches)**

BC_3_F_2_

t-Test: Two-Sample Assuming Unequal Variances

|  | Non-mutant | *rmr12-3* |
| --- | --- | --- |
| Mean | 8.625 | 2.058823529 |
| Variance | 8.116666667 | 1.933823529 |
| Observations | 16 | 17 |
| Hypothesized Mean Difference | 0 |  |
| df | 21 |  |
| t Stat | 8.332027005 |  |
| P(T<=t) one-tail | 2.13236E-08 |  |
| t Critical one-tail | 1.720742903 |  |
| P(T<=t) two-tail | 4.26472E-08 |  |
| t Critical two-tail | 2.079613845 |  |

BC_5_F_2_

t-Test: Two-Sample Assuming Unequal Variances

|  | Non-mutant | *rmr12-3* |
| --- | --- | --- |
| Mean | 6.75 | 1.142857143 |
| Variance | 1.071428571 | 0.142857143 |
| Observations | 8 | 7 |
| Hypothesized Mean Difference | 0 |  |
| df | 9 |  |
| t Stat | 14.27272727 |  |
| P(T<=t) one-tail | 8.67853E-08 |  |
| t Critical one-tail | 1.833112933 |  |
| P(T<=t) two-tail | 1.73571E-07 |  |
| t Critical two-tail | 2.262157163 |  |

**Figure 4E (Central spike branch length)**

BC_3_F_2_

t-Test: Two-Sample Assuming Unequal Variances

|  | Non-mutant | *rmr12-3* |
| --- | --- | --- |
| Mean | 24.4375 | 12.5882353 |
| Variance | 5.72916667 | 13.6323529 |
| Observations | 16 | 17 |
| Hypothesized Mean Difference | 0 |  |
| df | 28 |  |
| t Stat | 11.0018798 |  |
| P(T<=t) one-tail | 5.6139E-12 |  |
| t Critical one-tail | 1.70113093 |  |
| P(T<=t) two-tail | 1.1228E-11 |  |
| t Critical two-tail | 2.04840714 |  |

BC_5_F_2_

t-Test: Two-Sample Assuming Unequal Variances

|  | Non-mutant | *rmr12-3* |
| --- | --- | --- |
| Mean | 23.75 | 10.5714286 |
| Variance | 1.64285714 | 16.6190476 |
| Observations | 8 | 7 |
| Hypothesized Mean Difference | 0 |  |
| df | 7 |  |
| t Stat | 8.20540452 |  |
| P(T<=t) one-tail | 3.875E-05 |  |
| t Critical one-tail | 1.89457861 |  |
| P(T<=t) two-tail | 7.7499E-05 |  |
| t Critical two-tail | 2.36462425 |  |

**Figure 4F (No. ear shoots)**

BC_3_F_2_

t-Test: Two-Sample Assuming Unequal Variances

|  | Non-mutant | *rmr12-3* |
| --- | --- | --- |
| Mean | 2.25 | 1.05882353 |
| Variance | 0.46666667 | 0.43382353 |
| Observations | 16 | 17 |
| Hypothesized Mean Difference | 0 |  |
| df | 31 |  |
| t Stat | 5.09376878 |  |
| P(T<=t) one-tail | 8.2077E-06 |  |
| t Critical one-tail | 1.69551878 |  |
| P(T<=t) two-tail | 1.6415E-05 |  |
| t Critical two-tail | 2.03951345 |  |

BC_5_F_2_

t-Test: Two-Sample Assuming Unequal Variances

|  | Non-mutant | *rmr12-3* |
| --- | --- | --- |
| Mean | 2.875 | 1.14285714 |
| Variance | 0.41071429 | 0.14285714 |
| Observations | 8 | 7 |
| Hypothesized Mean Difference | 0 |  |
| df | 12 |  |
| t Stat | 6.46666667 |  |
| P(T<=t) one-tail | 1.5419E-05 |  |
| t Critical one-tail | 1.78228756 |  |
| P(T<=t) two-tail | 3.0838E-05 |  |
| t Critical two-tail | 2.17881283 |  |

t-Test: Two-Sample Assuming Unequal Variances

**Figure 5A**

| t-Test: Two-Sample Assuming Unequal Variances | | |
| --- | --- | --- |
|  | *Rmr12* | *rmr12-3* |
| Mean | 0.32657075 | 0.39889648 |
| Variance | 0.00109672 | 0.00071844 |
| Observations | 2 | 3 |
| Hypothesized Mean Difference | 0 |  |
| df | 2 |  |
| t Stat | -2.5767544 |  |
| P(T<=t) one-tail | 0.06167663 |  |
| t Critical one-tail | 2.91998558 |  |
| P(T<=t) two-tail | 0.12335327 |  |
| t Critical two-tail | 4.30265273 |  |

**Figure 6**

| t-Test: Two-Sample Assuming Unequal Variances | | |
| --- | --- | --- |
|  | *Rmr12* | *rmr12-3* |
| Mean | 0 | -3.4183333 |
| Variance | 1.1752861 | 1.88343333 |
| Observations | 3 | 3 |
| Hypothesized Mean Difference | 0 |  |
| df | 4 |  |
| t Stat | 3.3853628 |  |
| P(T<=t) one-tail | 0.0138241 |  |
| t Critical one-tail | 2.1318468 |  |
| P(T<=t) two-tail | 0.0276481 |  |
| t Critical two-tail | 2.7764451 |  |

**Supplemental Figure 3A (Viable pollen frequency)**

t-Test: Two-Sample Assuming Unequal Variances

|  | Non-mutant | *+ / rmr12-4* |
| --- | --- | --- |
| Mean | 0.971711233 | 0.9722411 |
| Variance | 5.18839E-05 | 0.00015423 |
| Observations | 4 | 4 |
| Hypothesized Mean Difference | 0 |  |
| df | 5 |  |
| t Stat | -0.07381454 |  |
| P(T<=t) one-tail | 0.47201 |  |
| t Critical one-tail | 2.015048373 |  |
| P(T<=t) two-tail | 0.94402 |  |
| t Critical two-tail | 2.570581836 |  |

**Supplemental Figure 3B (Pollen germination frequencies)**

t-Test: Two-Sample Assuming Unequal Variances

|  | Non-mutant | *+ / rmr12-4* |
| --- | --- | --- |
| Mean | 0.9827426 | 1 |
| Variance | 0.00991392 | 0 |
| Observations | 8 | 8 |
| Hypothesized Mean Difference | 0 |  |
| df | 7 |  |
| t Stat | -0.4902275 |  |
| P(T<=t) one-tail | 0.31948196 |  |
| t Critical one-tail | 1.89457861 |  |
| P(T<=t) two-tail | 0.63896393 |  |
| t Critical two-tail | 2.36462425 |  |

**Supplemental Figure 3C (Pollen tube lengths)**

t-Test: Two-Sample Assuming Unequal Variances

|  | *Wx1* | *wx1* |
| --- | --- | --- |
| Mean | 0.16202216 | 0.15712531 |
| Variance | 0.00384784 | 0.00365108 |
| Observations | 361 | 407 |
| Hypothesized Mean Difference | 0 |  |
| df | 750 |  |
| t Stat | 1.10525365 |  |
| P(T<=t) one-tail | 0.13470206 |  |
| t Critical one-tail | 1.64688785 |  |
| P(T<=t) two-tail | 0.26940412 |  |
| t Critical two-tail | 1.96313204 |  |

**Supplemental Table 8**

Cluster 1

| t-Test: Two-Sample Assuming Unequal Variances | | |
| --- | --- | --- |
|  | *Rmr12* | *rmr12-3* |
| Mean | 1.90111999 | 1.76054564 |
| Variance | 0.02029654 | 0.58037076 |
| Observations | 2 | 3 |
| Hypothesized Mean Difference | 0 |  |
| df | 2 |  |
| t Stat | 0.31153844 |  |
| P(T<=t) one-tail | 0.3924336 |  |
| t Critical one-tail | 2.91998558 |  |
| P(T<=t) two-tail | 0.78486721 |  |
| t Critical two-tail | 4.30265273 |  |

Cluster 2

| t-Test: Two-Sample Assuming Unequal Variances | | |
| --- | --- | --- |
|  | *Rmr12* | *rmr12-3* |
| Mean | 1.154209 | 1.73347388 |
| Variance | 0.23672344 | 0.02268355 |
| Observations | 2 | 3 |
| Hypothesized Mean Difference | 0 |  |
| df | 1 |  |
| t Stat | -1.6323934 |  |
| P(T<=t) one-tail | 0.17495293 |  |
| t Critical one-tail | 6.31375151 |  |
| P(T<=t) two-tail | 0.34990587 |  |
| t Critical two-tail | 12.7062047 |  |

Cluster 3

| t-Test: Two-Sample Assuming Unequal Variances | | |
| --- | --- | --- |
|  | *Rmr12* | *rmr12-3* |
| Mean | 0.48314894 | 0.93587512 |
| Variance | 0.00218508 | 0.15783712 |
| Observations | 2 | 3 |
| Hypothesized Mean Difference | 0 |  |
| df | 2 |  |
| t Stat | -1.9535683 |  |
| P(T<=t) one-tail | 0.09498567 |  |
| t Critical one-tail | 2.91998558 |  |
| P(T<=t) two-tail | 0.18997135 |  |
| t Critical two-tail | 4.30265273 |  |

Cluster 4

| t-Test: Two-Sample Assuming Unequal Variances | | |
| --- | --- | --- |
|  | *Rmr12* | *rmr12-3* |
| Mean | 1.26169024 | 0.9399857 |
| Variance | 0.48744303 | 0.05071017 |
| Observations | 2 | 3 |
| Hypothesized Mean Difference | 0 |  |
| df | 1 |  |
| t Stat | 0.63015769 |  |
| P(T<=t) one-tail | 0.32101447 |  |
| t Critical one-tail | 6.31375151 |  |
| P(T<=t) two-tail | 0.64202894 |  |
| t Critical two-tail | 12.7062047 |  |

Cluster 5

| t-Test: Two-Sample Assuming Unequal Variances | | |
| --- | --- | --- |
|  | *Rmr12* | *rmr12-3* |
| Mean | 0.96377658 | 1.00230217 |
| Variance | 0.17581309 | 0.01887022 |
| Observations | 2 | 3 |
| Hypothesized Mean Difference | 0 |  |
| df | 1 |  |
| t Stat | -0.1255254 |  |
| P(T<=t) one-tail | 0.46025192 |  |
| t Critical one-tail | 6.31375151 |  |
| P(T<=t) two-tail | 0.92050383 |  |
| t Critical two-tail | 12.7062047 |  |

Cluster 6

| t-Test: Two-Sample Assuming Unequal Variances | | |
| --- | --- | --- |
|  | *Rmr12* | *rmr12-3* |
| Mean | 1.07630041 | 1.28844107 |
| Variance | 0.33458525 | 0.15956926 |
| Observations | 2 | 3 |
| Hypothesized Mean Difference | 0 |  |
| df | 2 |  |
| t Stat | -0.4517904 |  |
| P(T<=t) one-tail | 0.34784372 |  |
| t Critical one-tail | 2.91998558 |  |
| P(T<=t) two-tail | 0.69568744 |  |
| t Critical two-tail | 4.30265273 |  |

Cluster 7

| t-Test: Two-Sample Assuming Unequal Variances | | |
| --- | --- | --- |
|  | *Rmr12* | *rmr12-3* |
| Mean | 1.59989611 | 2.29867913 |
| Variance | 0.16878552 | 0.0171286 |
| Observations | 2 | 3 |
| Hypothesized Mean Difference | 0 |  |
| df | 1 |  |
| t Stat | -2.3279541 |  |
| P(T<=t) one-tail | 0.12914727 |  |
| t Critical one-tail | 6.31375151 |  |
| P(T<=t) two-tail | 0.25829454 |  |
| t Critical two-tail | 12.7062047 |  |
